# Dissection of paracrine/autocrine interplay in lung tumor microenvironment mimicking cancer cell-monocyte co-culture models reveals proteins that promote inflammation and metastasis

**DOI:** 10.1186/s12885-023-11428-7

**Published:** 2023-10-02

**Authors:** Asif Amin, Aabid Mustafa Koul, Umer Majeed Wani, Faizah Farooq, Basit Amin, Zubair Wani, Asif Lone, Ayub Qadri, Raies A. Qadri

**Affiliations:** 1 Immunobiology Lab, Department of Biotechnology, University of Kashmir, Srinagar, J&K 190006 India; 2 Department of Biotechnology, All India Institute of Medical Sciences, New Delhi, 110608 India; 3 Abdul Kalam Chair for Translational Research, Islamic University of Science and Technology, Awantipora, J&K 192122 India

**Keywords:** Tumor microenvironment, Tumor associated macrophages, Inflammation, Secretome, Metastasis

## Abstract

**Background:**

Tumor cell-monocyte interactions play crucial roles in shaping up the pro-tumorigenic phenotype and functional output of tumor-associated macrophages. Within the tumor microenvironment, such heterotypic cell–cell interactions are known to occur via secretory proteins. Secretory proteins establish a diabolic liaison between tumor cells and monocytes, leading to their recruitment, subsequent polarization and consequent tumor progression.

**Methods:**

We co-cultured model lung adenocarcinoma cell line A549 with model monocytes, THP-1 to delineate the interactions between them. The levels of prototypical pro-inflammatory cytokines like TNF-𝛼, IL-6 and anti-inflammatory cytokines like IL-10 were measured by ELISA. Migration, invasion and attachment independence of lung cancer cells was assessed by wound healing, transwell invasion and colony formation assays respectively. The status of EMT was evaluated by immunofluorescence. Identification of secretory proteins differentially expressed in monocultures and co-culture was carried out using SILAC LC–MS/MS. Various *insilico* tools like Cytoscape, Reacfoam, CHAT and Kaplan–Meier plotter were utilized for association studies, pathway analysis, functional classification, cancer hallmark relevance and predicting the prognostic potential of the candidate secretory proteins respectively.

**Results:**

Co-culture of A549 and THP-1 cells in 1:10 ratio showed early release of prototypical pro-inflammatory cytokines TNF-𝛼 and IL-6, however anti-inflammatory cytokine, IL-10 was observed to be released at the highest time point. The conditioned medium obtained from this co-culture ratio promoted the migration, invasion and colony formation as well as the EMT of A549 cells. Co-culturing of A549 with THP-1 cells modulated the secretion of proteins involved in cell proliferation, migration, invasion, EMT, inflammation, angiogenesis and inhibition of apoptosis. Among these proteins Versican, Tetranectin, IGFBP2, TUBB4B, C2 and IFI30 were found to correlate with the inflammatory and pro-metastatic milieu observed in our experimental setup. Furthermore, dysregulated expression of these proteins was found to be associated with poor prognosis and negative disease outcomes in lung adenocarcinoma compared to other cancer types. Pharmacological interventions targeting these proteins may serve as useful therapeutic approaches in lung adenocarcinoma.

**Conclusion:**

In this study, we have demonstrated that the lung cancer cell-monocyte cross-talk modulates the secretion of IFI30, RNH1, CLEC3B, VCAN, IGFBP2, C2 and TUBB4B favoring tumor growth and metastasis.

**Supplementary Information:**

The online version contains supplementary material available at 10.1186/s12885-023-11428-7.

## Introduction

Tumors are highly complex tissues consisting of malignant cells besides a variety of stromal cell populations, all of which act in concert to propel tumor growth and metastasis. Tumor-infiltrating macrophages (TIMs) are recruited from a circulating pool of monocytes and once present in the vicinity of the tumor cells are re-educated for a phenotype promoting tumor growth and metastasis and are accordingly, referred to as Tumor-associated macrophages (TAMs). TAMs constitute the predominant stromal cells populating solid tumors and support tumor growth and facilitate metastatic dissemination [1, 2]. There are considerable clinical studies that demonstrate a strong correlation between a high population of TAMs and poor prognosis or survival in lung, breast, ovarian, and cervical cancers [3–6]. However, the molecular mechanisms by which tumor cells interact with macrophages and re-educate them for a phenotype beneficial for tumor progression and metastasis remains poorly defined. A symbiotic relationship between tumor cells and TAMs has been proposed wherein tumor derived molecules attract TAMs, promote their survival and in reciprocation receive signals and factors that promote tumor progression and metastasis [7].

Secretory proteins have long been studied to act as a source of communication among various cell types in tumor microenvironment [8]. Considering the key role of such proteins in mediating intercellular interactions, it can therefore be envisaged that the secretory proteins establish a diabolic liaison between tumor cells and TAMs, leading to monocyte recruitment, their subsequent polarization and consequent tumor progression. Analysis of the secretome, therefore offers an attractive approach to elucidate the mechanism driving interactions between cancer cells and the TAMs. Although a few studies have been conducted to screen the cancer cell secreted factors that activate macrophages [9], none of the studies have comprehensively carried out the secretome profiling of human cancer cell-monocyte microenvironmnent. In this context, the present study was designed and a SILAC based quantitative proteomic approach was adopted to identify these secretory proteins in tumor microenvironment simulating co-culture models involving model human lung adenocarcinoma cell line A549 and monocytic cell line, THP-1. We demonstrated that the interaction between lung carcinoma cells and monocytes is a two-way process and involves cancer cell-derived proteins that are capable of modulating the monocytes to generate a milieu congenial for metastatic growth in vitro.

## Materials and methods

### Cell culture

Human lung adenocarcinoma cell line, A549 and human monocytic leukemia cell line, THP-1 used in the study were purchased from National Centre for Cell Science, Pune, India. Cell line authentication at the repository was carried out by short tandem repeat (STR) profiling. A549 and THP-1 cells were maintained in DMEM and RPMI‐1640 respectively supplemented with 10% FBS and 100 U/ml penicillin. Cells were cultured under 5% CO2 and 37 °C temperature [10].

### Cytokine quantification

TNF-α, IL-6 and IL-10 levels were quantified from culture supernatants using commercially available kits following manufacturer’s instructions. Briefly, a 96-well microplate (Maxisorp, Genetix) was coated with 50 µl capture antibody (diluted 1: 250 in 100mM carbonate buffer, pH 9.5) and kept overnight at 4 °C. Then plate was washed 3 times with PBS-Tween (PBST) and blocked with PBS-BSA-1% (100 µl/well) for 1 h at 37 °C. After washing, samples were added to each well and the plate was incubated for 1 h at 37 °C. Subsequently, the plate was washed and incubated with detection reagent mix (detection antibody + avidin-HRP) diluted 1: 250 in PBS-BSA 1%. After 1 h of incubation, the plate was washed and the enzyme activity was determined by adding freshly prepared substrate solution containing TMB/TBABH/H_2_O_2_ (50 µl/well). The reaction was stopped with 50 µl of 2 N H_2_SO_4_ and the absorbance was read at 450 nm (or as advised in the manufacturer’s instructions). All the assays were performed in triplicate.

### Migration assay

A549 cells were plated in 30 mm dishes and grown upto 90% confluency in DMEM supplemented with 10% FBS. The media was then removed and the monolayer was scratched with a 200 ml pipette tip, washed twice with PBS to remove detached cells and photographed (t = 0). Cells were then incubated in co-culture conditioned medium or homotypic conditioned medium for 24 h. Then the wounds were observed and photographed (t = 24). The assay was repeated three independent times. The percentage of wound closure was estimated as reported in [11].

### Invasion assay

The polycarbonate filter inserts (8 mm pore size, Corning) precoated with Matrigel were pre-incubated in DMEM supplemented with 1% FBS for 2 h before the cells were plated. A549 cells (5 × 10^4^) in 200 µl of co-culture conditioned medium or homotypic conditioned medium were seeded in the upper chamber. Then 500 µl DMEM supplemented with 10% FBS was added to the lower chamber as a chemotactic agent. After 24 h incubation, non-migrating cells in the upper chamber of the filters were removed using cotton swabs. The cells that migrated and adhered to the other side of the filter were fixed in 3.7% formaldehyde for 20 min, stained with crystal violet and counted per five fields.

### Soft agar colony formation assay

Anchorage independent growth in soft agar was used to assess the tumorigenic potential imparted by secretory Fibronectin and its associated EDA in vitro. The soft agar assay was performed in 6-well plates containing two layers of Agar. The bottom layer consisted of 0.8% agar in 1 ml of DMEM supplemented with 10% FBS. A549 cells (1 × 10^4^/well) were placed in the top layer containing 0.4% agar in the same medium as the bottom. Cells were cultured for 14 days under different conditions and colonies were photographed and counted per four fields under a microscope.

### Western blot

The samples to be analyzed were separated on 10% or 12% SDS-PAGE and transferred to a PVDF membrane by semi-dry blotting for 1.5 h at a constant current of 250 mA, using a semi-dry transfer apparatus (Siplast, UK). The transfer of proteins was ascertained by staining the PVDF membrane with Ponceau-S (1X). The membrane was blocked overnight at 4ºC with 1% BSA prepared in PBS and subsequently probed with the appropriate primary antibody at recommended dilutions and time points, followed by three washings with PBST. Finally the membrane was incubated in HRP or IR-labeled labeled secondary antibody for 1 h. After three washings with PBST, the membrane was developed using Enhanced Chemiluminescence reagents or scanned on an Infrared imager, Odyssey Licor (Licor Biosciences, USA).

### Immunofluorescence

A549 cells were seeded in 24 well tissue culture plates and grown overnight in DMEM supplemented with 10% FBS. After washing with serum free medium, cells were incubated in conditioned medium containing Fibronectin or medium supplemented with recombinant EDA for 24 h. Then the cells were fixed with 4% formaldehyde and permeabilized with PBS containing 0.1% Triton X-100 for 30 min. This was followed by blocking with 1% BSA in PBS for 1 h and incubation in primary antibody overnight (or as recommended) at 4 °C. After washings with PBST three times, cells were incubated in secondary anti-mouse IgG antibody conjugated with Alexa Fluor for 1 h, observed under Evos cell imaging system (Life Technologies, USA) and photographed.

### Quantitative real-time PCR

Total RNA was isolated from A549 and THP-1 cells as reported in [12]. Real-time PCR was performed using gene specific primers and SYBR Green (Kapa Biosystems, SA).The reaction mixture was run in lightCycler 480-II (Roche, Germany) and the Gene expression was quantified using 2^−ΔΔCT^ method [13].

### Labeling with stable isotopes

For stable isotopic labeling, DMEM was supplemented with stable heavier isotopes (_13_C^6^ L-Arginine[R]-HCl and _13_C^6^ L-Lysine[K]-2HCl) and RPMI with corresponding lighter isotopes (regular _12_C^6^ L-arginine[R] and _12_C^6^ L-lysine[K]) (Thermo Scientific Rockford, USA) at 0.1 g/L concentration in accordance with the SILAC kit instructions. A549 cells were cultured in the DMEM and THP-1 in RPMI containing heavier and lighter isotopes of L-Arginine and L-Lysine respectively. In order to achieve approximately 99% incorporation of the heavy and light isotopes into the proteins, A549 and THP-1 were repeatedly sub-cultured for ~ 7 passages (~ 5 weeks). Repeated seeding of cells marked as AhdD10 and TldR10 grown in T-75 flasks was performed. Every other day, the cells were supplemented with labeled media. A549 cells were dislodged by Enzyme-free Cell Dissociation Buffer (Invitrogen Grand Island, USA) after achieving confluence, whereas THP-1 cells were centrifuged for 5 min at 800 g. After the adaptation phase, when cells reached around 80% confluence, they were handled individually by centrifuging and washing them thrice using RPMI devoid of Arginine (R) and Lysine (K). Finally, the cells were sorted into three different categories: A549 monoculture (1 × 10^6^), THP-1 monoculture (10 × 10^6^), and a co-culture mix of 1:10 ratios (1 × 10^6^ [A549]: 10 × 10^6^ [THP-1]) and were cultured for 48 h in RPMI (lacking arginine and lysine).

### Collection and processing of the Secretome

Conditioned media from each of the three categories were harvested after 48 h and centrifuged for 30 min at 800 g to remove any floating and unattached cells. In order to remove the cellular debris, the supernatants were again filtered by running through a Millex-GP 0.22 μm filter (Millipore, Ireland). Following this, filtered secretomes were concentrated using a 3 kDa cut-off Millipore Amicon Ultra filters (Millipore, Billerica, MA, USA) by centrifugation at 4000 g till the secretome was condensed to 1mL, and subsequently centrifuging at 14,000 g using the 3 kDa cut-off filters till a condensed volume of 500µL was obtained. Pierce microBCA kit (Thermo Scientific) was used to determine the protein concentration.

### Preparation of samples and trypsin (in-solution) digestion

Before MS analysis, each sample was subjected to in-solution digestion. 40 µg protein was lyophilized per sample and resuspended in 100mM TEABC (pH = 8.0). After being reduced using 5mM dithiothreitol at 60℃ for 30 min, the samples were alkylated at room temperature with 20mM iodoacetamide. Proteins were digested at 37 °C for 16 h with an enzyme – protein ratio of 1:20 (w/w) utilizing sequencing grade trypsin (modified sequencing grade; Promega, Madison, WI). Peptides were dried in a vacufuge concentrator after trypsin digestion, desalted with C18 Stage Tips, and kept at -80 °C till LC-MS/MS analysis could be performed.

### LC-MS/MS

Digested samples were inspected by LC-MS/MS analysis using an Orbitrap Fusion Tribrid mass spectrometer (Thermo Scientific, Bremen, Germany) coupled to an Easy nLC-1000 system (Thermo Scientific, Odense, Denmark). The already digested peptides were first reconstituted in 0.1% formic acid before being mounted into a trap column (75 μm × 2 cm; 3 μm C18 100 A˚, Thermo Scientific, Acclaim Pepmap 100) utilizing solvent A (i.e., 0.1% formic acid) and then resolved on an analytical column (75 μm × 50 cm; 2 μm C18 100 A˚, Thermo Scientific, Acclaim PepMap RSLC). For peptide resolution, a linear gradient spanning 10–32% of solvent B (i.e., 0.1% formic acid in 95% acetonitrile) was run at a flow rate of 300 nl/min for 105 min (total run time: 120 min). The data was retrieved using the data-dependent acquisition approach, with a scan spectrum ranging between 400 and 1600, a mass resolution of 120,000, a maximum injection duration of 50 ms, and an AGC target of 2 × e^6^ ions. Utilizing an isolation window adjusted at 1.6 m/z in a quadrupole mass filter, the top ten precursor ions were separated. To facilitate the dissociation of the ions in the precursor pool, they were exposed to greater energy collision-induced dissociation with 34% normalised collision energy. The MS/MS scans were generated at a maximal ion injection time of 200 ms, with a mass resolution of 30,000, as well as an AGC target of 1 × e^6^ ions. In order to have mass measurement precision, the polydimethylcyclosiloxane (m/z, 445.1200025) ion was utilized, and the lock mass was enabled. Independent triplicate runs of each sample were performed on an Orbitrap Fusion mass spectrometer.

### Analysis of LC-MS/MS data

Raw data generated from LC-MS/MS was investigated using proteome discoverer [PD] (Version 2.1.1) software (Thermo Fisher Scientific, Bremen, Germany) employing the Mascot and Sequest search engine algorithms when compared to the Human refseq-81 database. Carbamidomethylation at the cysteine residue was used as a static modification, whereas oxidation of methionine and SILAC (C6-arginine/lysine) were used as variable modifications in the database search variables. For both the precursor and the fragment ions, a mass error of 10 ppm and 0.05 Da, respectively, was permitted. Trypsin, a designated protease, and a single missed cleavage was considered acceptable. The false discovery rate (FDR) was calculated by carrying out decoy database searches and FDR cut-off for peptide and protein identification was set to 0.01. For quantification, exclusively unique peptides were taken into account. Peptide Spectrum Match (PSM) entries with associated peak regions were exported from PD. For the co-culture setting, the PSM entry was divided up into two independent files: heavy and light, which corresponded to heavy-labelled cancer cells and light-labelled monocytes, respectively. Peptide and protein lists were created using an in-house tool. For further analysis those proteins were further taken into consideration that were present in at least two of the three replicates. Protein normalization for individual cell lines between mono-culture and their corresponding co-culture (e.g. A549 mono & A549 co-culture) was done based on summed peak area of all the proteins. The normalization factor was calculated by taking the ratio of total peak area in mono and co-culture. Proteins ascertained from each cell line in a monoculture scenario were compared with those ascertained in a co-culture scenario (i.e., A549 monoculture versus A549 co-culture; THP-1 monoculture versus THP-1 co-culture). Using these two comparative sets of data, three protein lists were generated: (i) Proteins that were exclusively found in A549 and THP-1 mono-cultures, respectively, (ii) Proteins that were exclusively found in A549 and THP-1 co-cultures, respectively and (iii) Proteins that were found in both mono- and co-culture scenarios. Fold change was computed for the third group of proteins by evaluating the area of each proteins in mono- as well as co-culture scenarios. Identified proteins that have at least two unique peptides and four PSMs with a %CV of ≤ 30 are documented.

### Bioinformatics analysis

Several bioinformatics algorithmic methods were used for shortlisting and ascertaining the candidature of the identified proteins [14]. JVenn was employed across all six scenarios to determine which proteins were unique and which were shared among the groups. Morpheus, a versatile matrix visualization and analysis software [15] was employed to generate the heatmaps. With the help of Morpheus, heat map of all the proteins in six different scenarios were generated firstly based on %CV of < 10 and/or fold-change of > 3, and then secondly based on SecretomePv2.0 [16] and SignalPv4.1 [17] respectively… To further illustrate the function of all secretory proteins in each scenario, Cytoscapev3.8.2 platform was utilized and the software packages STRINGv8.3 to display the interaction of narrowed down secretory proteins across and within the different scenarios. BiNGOv3.0.5 (Biological Networks Gene Ontology) was used to visualize the involvement of the narrowed down secretory proteins. STRING (Search Tool for the Retrieval of Interacting Genes/Proteins) is a repository of protein interactions, both established and presumed whereas BiNGO integrates the prominent functional features of a particular gene/protein set onto the GO hierarchical order and produces same data as a Cytoscape diagram. To depict the connection between proteins and their respective pathways, tool bundle in the Reactomev79 was used. Reactome offers easy bioinformatics tools for pathway viewing, inference and analysis, e.g., molecular interactions between nucleic acids, proteins, complexes and small compounds [18]. The cancer hallmarks analytics tool [CHAT] [19](http://chat.lionproject.net/) is a text-mining assessment of several millions of PubMed articles that enables users to rapidly and simply examine the degree of correlation between gene/protein of interest and cancer hallmarks across literatures. CHAT was implemented to assess the shortlisted secretory proteins in light of previously recognized cancer hallmarks based on scientific literature.

## Results

### Lung cancer cells stimulate monocytes to release pro-tumorigenic cytokines

**Fig. 1:**
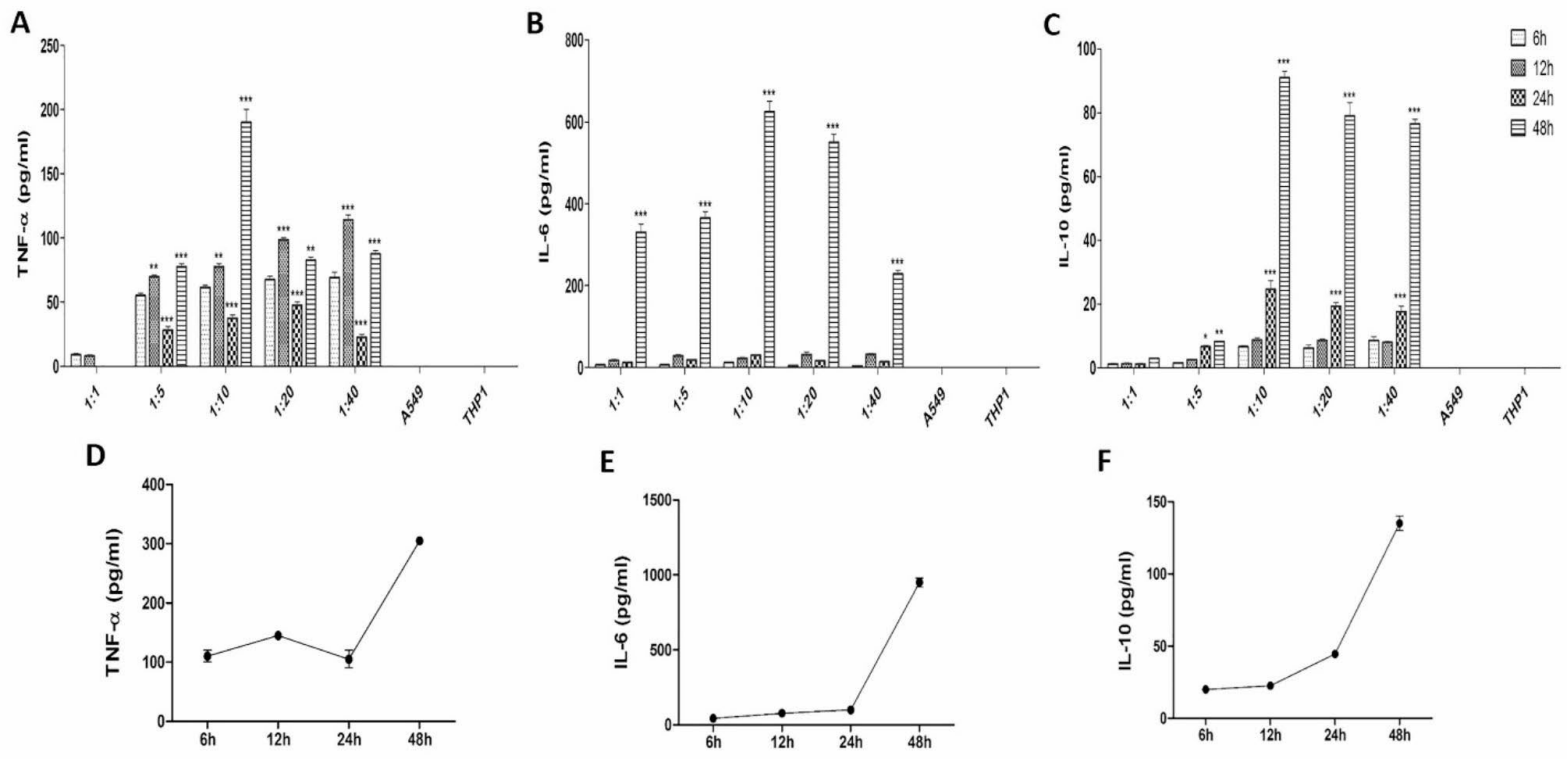
**Co-culture of Lung cancer cells and monocytes induces the release of TNF-α, IL-6 and IL-10 from THP-1 cells**. A549 cells were co-cultured with THP-1 cells in the selected ratios of 1:1, 1:5, 1:10, 1:20 and 1:40 for various time points and the co-culture conditioned medium was immunoassayed for **(A)** TNF-α **(B)** IL-6 and **(C)** IL-10. Expression pattern of **(D)** TNF-α, **(E)** IL-6 and **(F)** IL-10 from THP-1 cells after stimulation with 48 h A549 conditioned medium. Data represented as mean ± SD of results obtained from at least three independent experiments. *p < 0.05; **p < 0.01; ***p < 0.001 compared to control using Bonferroni Post-test

In order to simulate the interaction between tumor cells and monocytes in tumor microenvironment, human model lung carcinoma cells, A549 and model monocytic cells, THP-1 were co-cultured in varying ratios of 1:1, 1:5, 1:10, 1:20 and 1:40, respectively, for various time points including 6 h, 12 h, 24 and 48 h. After co-culture, conditioned medium was harvested and levels of prototypical pro-inflammatory cytokines like TNF-𝛼, IL-6 and anti-inflammatory cytokine like IL-10 were measured by enzyme-linked immunoassay. All the tested co-cultures showed significant production of TNF-𝛼, and IL-6, however the most potent release of these cytokines was observed at co-culture ratios of 1:10 (Fig. 1A and B). The levels of these cytokines was observed to increase with time with response peaking at 48 h time point. The expression kinetics of IL-10 however showed a distinct pattern, although the most potent release was observed at the co-culture ratio of 1:10. IL-10 was detected only after 24 h of co-culture with the maximum release being observed at 48 h (Fig. 1C). The release of these cytokines followed similar expression kinetics when THP-1 cells were incubated in the conditioned medium obtained from A549 cells at 48 h time point, with maximum release of TNF-𝛼, IL-6 and IL-10 observed at 48 h (Fig. 1D and E F). These results, thus indicate that a stimulus, not only cell associated but also released from A549 cells activates the THP-1 cells. Intriguingly, neither A549 nor THP-1 cultures alone produced any of these cytokines.

### Lung cancer cell-monocyte interaction milieu promotes migration, invasion, and attachment independence of lung cancer cells

**Fig. 2:**
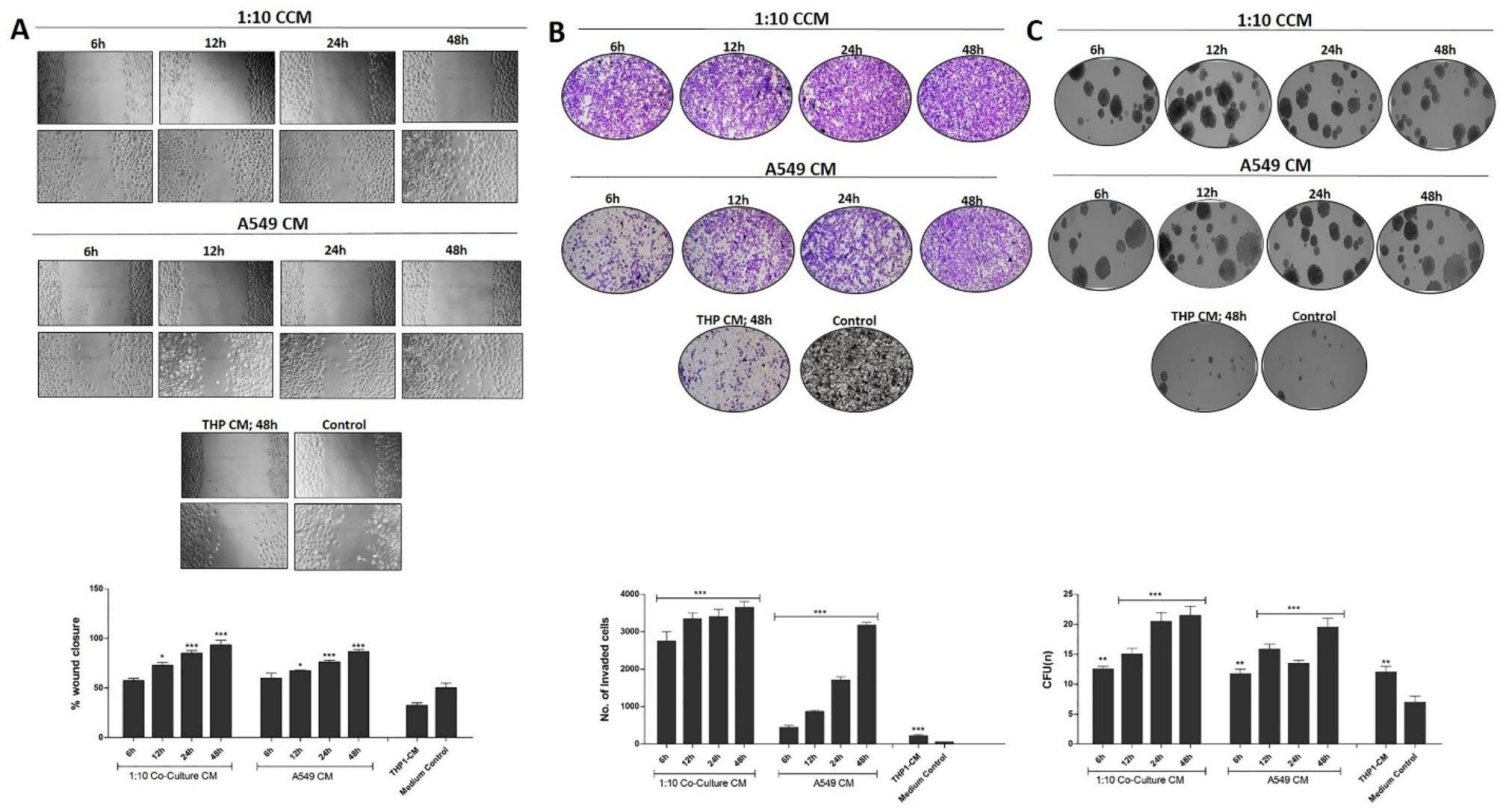
**Co-culture conditioned medium promotes migration, invasion, and attachment independent growth of lung cancer cells**. **(A)** A549 cells were grown to sub-confluency in 30 mm culture dishes, scratched with sterile tip and incubated in conditioned medium obtained from various co-culture or homotypic culture conditions. Scratched areas were photographed at zero hour (T = 0) and then subsequently later at 24 h (T = 24). The scratched areas were quantified in three random fields in each case, and data were calculated from at least three independent experiments and represented as mean ± SD. *p < 0.05; **p < 0.01; ***p < 0.001 compared to control using Bonferroni Post-test. **(B)** A549 cells were seeded in matrigel coated transwell chambers containing conditioned medium obtained from various co-culture or homotypic culture conditions and allowed to invade. After 24 h, the invaded cells were fixed, stained, quantified and photographed. Data represented as mean ± SD of results obtained from at least three independent experiments. *p < 0.05; **p < 0.01; ***p < 0.001 compared to control using Bonferroni Post-test. **(C)** A549 cells were seeded in the top layer containing 0.4% agar and cultured for 14 days in conditioned medium obtained from various co-culture or homotypic culture conditions. Colonies were photographed and counted per four fields under a microscope. The number of colonies was counted and the data was interpreted as CFU. Data represented as mean ± SD of results obtained from at least three independent experiments

Since 1:10 ratio of co-culture was most effective in effectuating the release of cytokines at all the time points tested, we next tried to evaluate the effect of 1:10 co-culture conditioned medium on the metastatic behavior of A549 cells. A549 and THP-1 cells were co-cultured in 1:10 ratio for different time points to collect the conditioned medium. To investigate whether the co-culture conditioned medium promotes the cell migration, A549 cells were grown to sub-confluency (70–80%), wounded and then cultured in co-culture conditioned medium (CCM) or homotypic conditioned medium (CM) obtained from monoculture of A549 cells or THP-1 cells and non-conditioned medium (control). After 24 h of incubation it was observed that A549 cells treated with 48 h co-culture conditioned medium showed almost complete obliteration of the wounded area, although 24 h, 12 h and 6 h co-culture conditioned medium proved to be effective as well (Fig. 2A). However, a noteworthy observation was the closure of wound by treatment with homotypic conditioned medium obtained from A549 cells, with 48 h conditioned medium being most effective followed by 24 h, 12 h and 6 h. This effect of the homotypic conditioned medium was however, less pronounced than the co-culture medium. Cells treated with homotypic conditioned medium obtained from THP-1 cells or unconditioned medium (medium control) showed marginal healing. Subsequently, we performed an in vitro invasion assay to determine whether co-culture conditioned medium could promote invasiveness of A549 cells. A549 cells in co-culture or homotypic conditioned medium were seeded in the upper chamber of matrigel coated transwell insert with chemoattractant in the lower chamber. It was observed that A549 cells treated with 48 h co-culture conditioned medium showed maximal invasion across matrigel coated membrane of the insert when compared with 24 h, 12 h and 6 h co-culture conditioned medium, thus pointing to the secretion of soluble mediators from either or both the cell types during their reciprocal interactions in co-culture (Fig. 2B). A similar but less marked effect was seen when cells were seeded in homotypic conditioned medium obtained from A549 cells, with 48 h culture medium being most effective followed by 24 h, 12 h and 6 h. Treatment with homotypic conditioned medium obtained from THP-1 cells showed no obvious effects. Furthermore, we tested whether co-culture conditioned medium has any effect on tumorigenic potential of A549 cells. Soft agar assay colony formation assay was employed and A549 cells were grown under attachment independent conditions in presence of co-culture conditioned medium or homotypic conditioned medium for 14 days. It was observed that, A549 cells grown in presence of co-culture conditioned medium formed maximum number of colonies when compared to homotypic conditioned media or the control medium with kinetics akin to those observed in migration and invasion assays (Fig. 2C). These findings thus suggest that the reciprocal interactions between A549 and THP-1 cells result in the secretion of factors from either or both the cell types that in turn promote the metastatic properties of A549 cells. It is also explicit that A549 cells themselves secrete certain factors that augment their metastatic attributes in an autocrine manner.

### Lung cancer cell-monocyte interaction milieu promotes epithelial-mesenchymal transition in lung cancer cells

**Fig. 3:**
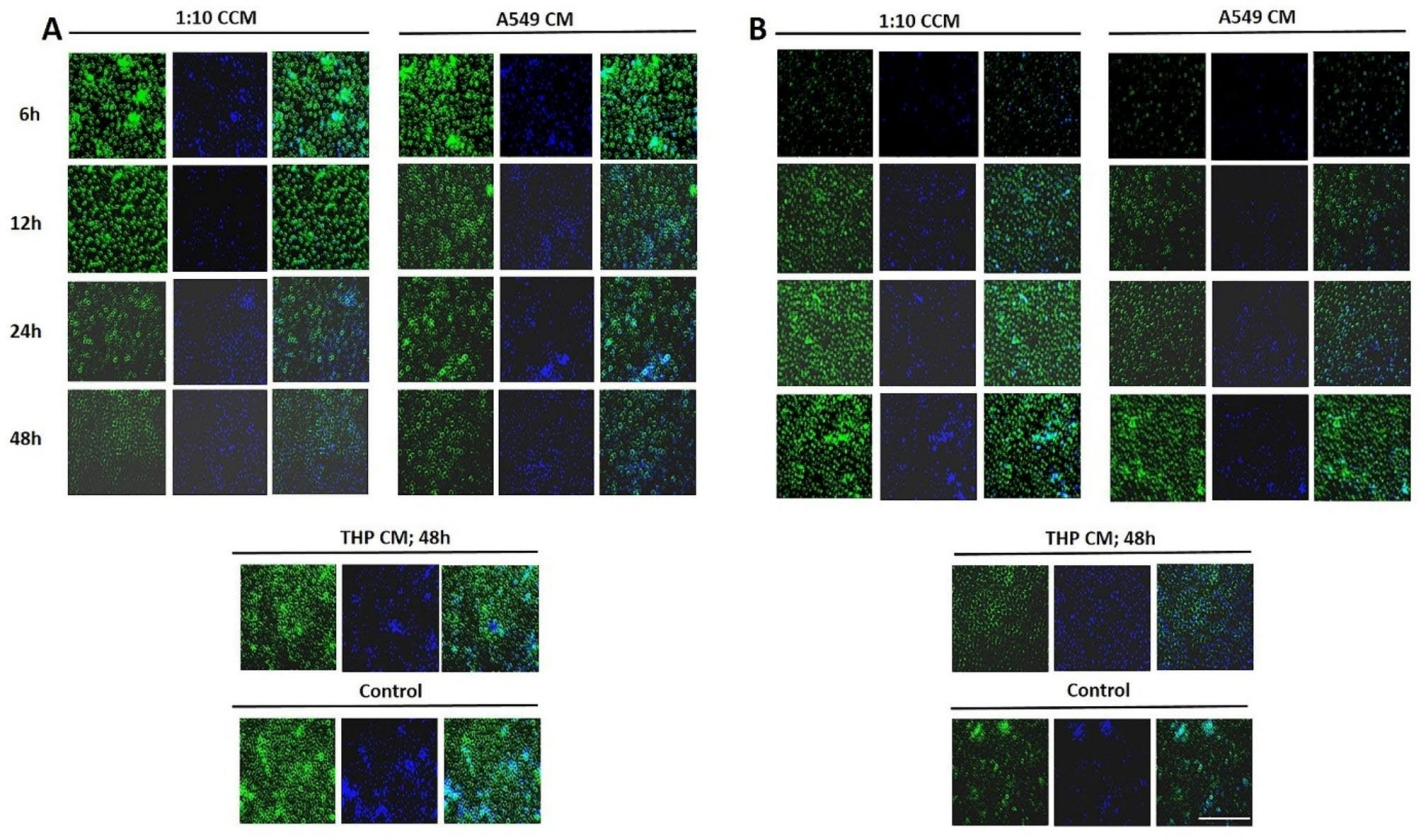
**Co-culture conditioned medium promotes EMT in lung cancer cells**. A549 cells were grown in conditioned medium obtained from various co-culture or homotypic culture conditions followed by immunofluorescence staining to assess the expression of **(A)** E-cadherin and **(B)** Vimentin using relevant primary antibody and alexa fluor labeled secondary antibody. Scale bar, 100 μm

Epithelial–mesenchymal transition (EMT) is a biological process fundamental to cell migration and invasion. Cancer cells often receive signals from their microenvironment which triggers their EMT. To substantiate the involvement of factors released due to lung cancer cell-monocyte cross-talk in promoting the metastatic properties of lung cancer cells, we assessed the expression of signature EMT markers in A549 cells under various culture conditions by immunofluorescence. It was observed that there was a progressive decrease in the expression of E-Cadherin and a concomitant increase in the expression of Vimentin when the cells were grown in 6 h, 12 h, 24 and 48 h co-culture conditioned medium respectively (Fig. 3). Similar albeit less conspicuous kinetics in the expression of E-Cadherin and Vimentin were observed under homotypic conditions. These results thus corroborate the findings that the reciprocal interactions between lung carcinoma and monocytes promote the migration, invasion and attachment independence of lung carcinoma cells.

### SILAC and conditioned medium obtention

**Fig. 4:**
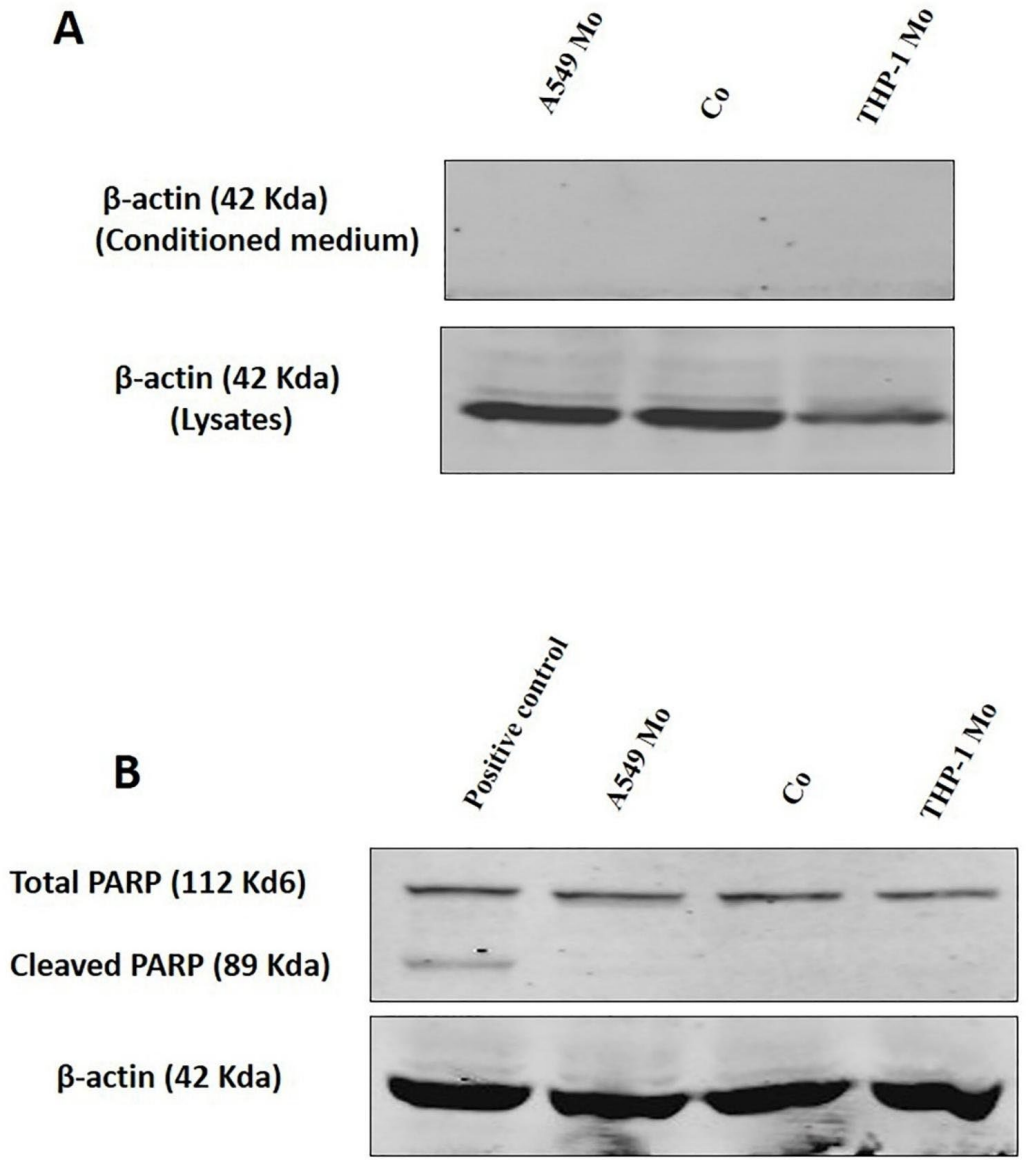
**Lung cancer cells and monocytes do not exhibit cell death in co-culture.** (A) Western blotting analysis indicating the expression of β-actin in the conditioned media and protein lysates obtained from A549 and THP- cells in monoculture and co-culture conditions. (B) Western blotting analysis indicating the cleavage status of PARP-1 in protein lysates obtained from A549 and THP-1 cells in monoculture and co-culture conditions. PARP-1 cleavage in control was induced by treatment of cells with 8 µM of doxorubicin. Full length blots are provided in supplementary Figure S2

In order to unravel the proteins mediating the reciprocal interactions between A549 and THP-1 cells in co-culture, a SILAC based quantitative proteomics strategy was employed. After labeling of A549 and THP-1 with heavy and light forms of arginine and lysine respectively, co-culturing of the two cell types in the 1:10 ratio (A549:THP-1) was carried out for 48 h. The work flow of the strategy followed is shown in the Figure S1. The conditioned media collected after the co-culture were designated as Co-CM while as that collected from monocultures of A549 cells or THP-1 (equal numbers to that used in co-culture) was designated as A549 Mo-CM and THP-1 Mo-CM respectively. To rule out the possibility of presence of intracellular proteins in the conditioned medium due to the leakage resulting from cell death, the presence of β-actin in conditioned medium was evaluated. Western blotting analysis of all the conditioned media revealed the absence of β-actin whereas the corresponding cell lysates were positive for β-actin (Fig. 4A). The possibility of activation of apoptotic programme in the two cell types due to co-culture was also assessed. In this direction, the cleavage status of PARP-1, one of several known cellular substrates of caspases was checked. Western blotting of the PARP-1 revealed the presence of intact 112 kda protein in all the conditions (Fig. 4B). These findings thus exclude cell death as an underlying reason for presence of protein(s) in conditioned media.

### Identification of secretory proteins differentially expressed in monocultures and co-culture

**Fig. 5:**
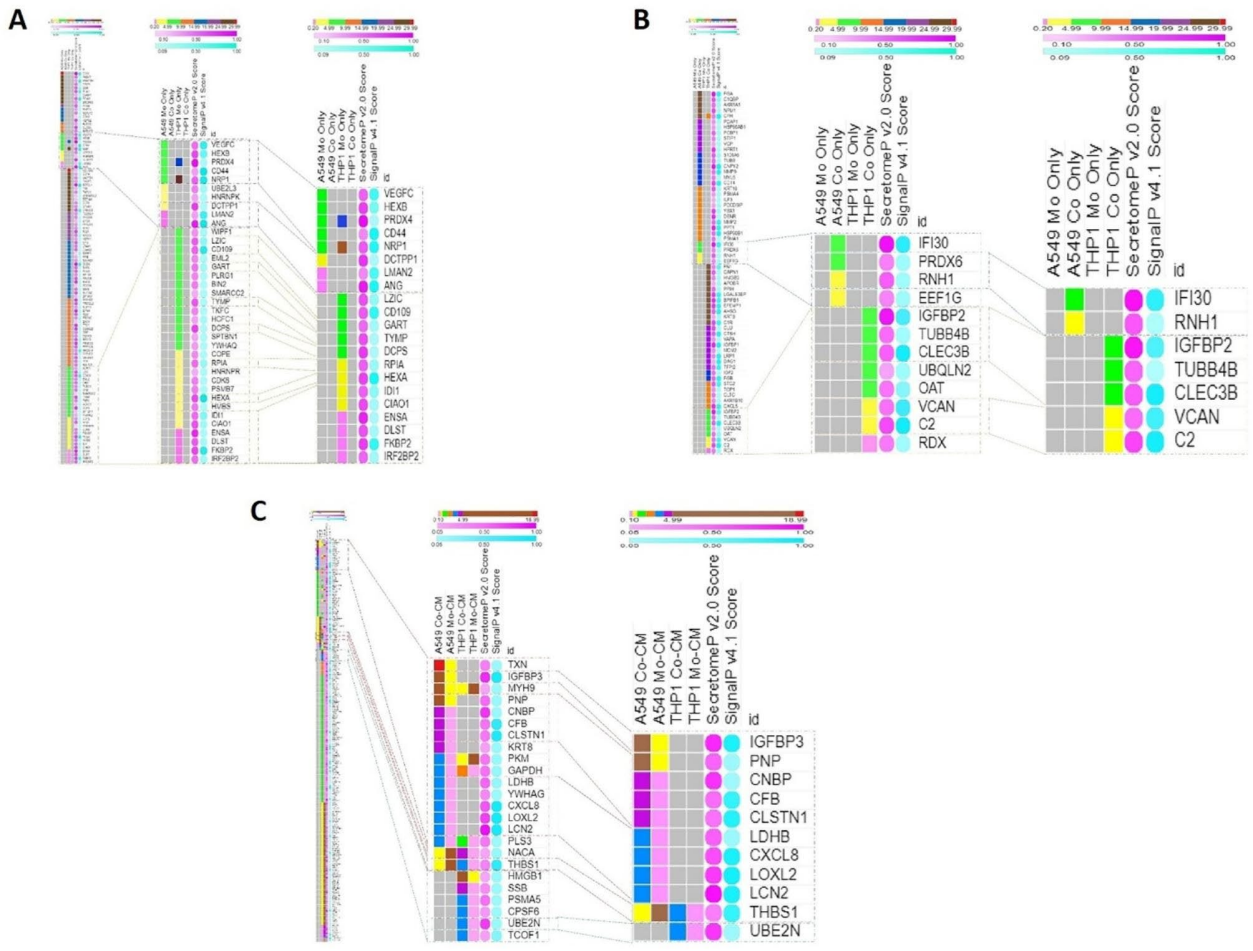
**Heat Map based on Versatile matrix visualization and analysis software, Morpheus. (A)** compares the total secretome protein profile of A549 Mo-CM and THP-1 Mo-CM based on %CV from > 30.0 to < 0.99 and their SecretomeP and SignalP scores (Left) with that of the actual secretory proteins based on SecretomeP (Non-classical secretory proteins) and SignalP (Classical Secretory Proteins) (Right) **(B)** compares the total secretome protein profile of A549 Co-CM and THP-1 Co-CM based on %CV from > 30.0 to < 0.99 and their SecretomeP and SignalP scores and their SecretomeP and SignalP scores (Left) with that of the actual secretory proteins based on SecretomeP (Non-classical secretory proteins) and SignalP (Classical Secretory Proteins) (Right) **(C)** compares the total secretome protein profile of A549 Co/Mo-CM and THP-1 Co/Mo-CM based on fold change from < 0.5 to > 5.0 (Left) with that of the actual secretory proteins based on SecretomeP (Non-classical secretory proteins) and SignalP (Classical Secretory Proteins) (Right)

The conditioned media samples were subjected to in-solution digestion followed by LC/MS-MS. A total of 414 proteins were identified and quantified in at least three biological replicates. Only proteins identified with at least 2 unique peptides and 4 PSMs with CV of ≤ 30% are reported. The details of protein identification and quantification are shown in Supplementary Datasheet 1 and Datasheet 2. Proteins found in mono-culture condition of each cell line were compared with proteins found in the corresponding co-culture condition (i.e., A549 mono Vs A549 co-culture and THP-1 mono vs. THP-1 co-culture). From each comparison three protein lists were obtained: (i) Proteins detected only in mono-culture condition (ii) Proteins detected only in co-culture condition (iii) Proteins showing the differential expression in both mono as well as co-culture condition. For the third set of proteins, fold change was calculated by comparing area of individual proteins between mono-culture and co-culture. Therefore a total of 6 scenarios were under study for the two cell types. Of the identified A549 cell proteins, 27 were specifically identified in monoculture (A549 Mo-CM) and 31 in co-culture (A549 Co-CM) whereas 59 proteins were found to be differentially expressed in co-culture (A549 Co/Mo CM). With respect to THP-1 cell proteins, 84 were specifically expressed in monoculture (THP-1 Mo-CM) and 35 in THP-1 co-culture (THP-1 Co-CM) whereas 178 THP-1 cell proteins were found to be differentially expressed in co-culture (Figure S3A). To assemble the proteins based on the range % CV and range fold-change, we arranged them into two groups and six sub-groups; four based on range % CV and two based on range fold-change (Figure S3B). (Figure S3B). Among the differentially expressed A549 proteins, 41 were upregulated and 18 were downregulated respectively in A549 Co/Mo CM scenario. For THP-1, 96 proteins were upregulated and 82 down regulated in co-culture. To further refine the data, proteins with %CV of < 10 were selected which led to the shortlisting of 10 proteins in case of A549 Mo-CM, 27 in THP-1 Mo-CM, 4 in A549 Co-CM and 8 in THP-1 Co-CM scenarios. Whilst among the differentially expressed proteins, only proteins with fold change of > 3 were further selected which led to shortlisting of 16 and 8 proteins in A549 Co/Mo CM and THP-1 Co/Mo CM scenarios respectively. This initial filtering based on %CV and fold-change led to the selection of a total of 73 proteins across all the scenarios. To investigate the secretory nature (classical or non-classical mode of secretion) of these proteins, neural-network probability algorithmic tools, such as SecretomeP and SignalP were employed. A protein is termed non-classically secreted if its neural network value is less than or equal to a specific threshold (NNscore ≥ 0.5) [16]. On the contrary, SignalP was used to determine if a protein is secreted in a classical manner. Classically secreted proteins are those that have obtained or exceed a certain limit of a likely signal peptide (D cut-off score ≥ 0.45) [20]. Overall, these criteria led to shortlisting of 39 secretory proteins from the initial selection of 73 proteins across all the groups, 20 (51.28%) of which were classical secretory proteins (CSPs) while 19 (48.72%) were non-classical secretory proteins (NCSPs) (Fig. 5).

### Association study, pathway analysis and functional classification of candidate secretory proteins

**Fig. 6:**
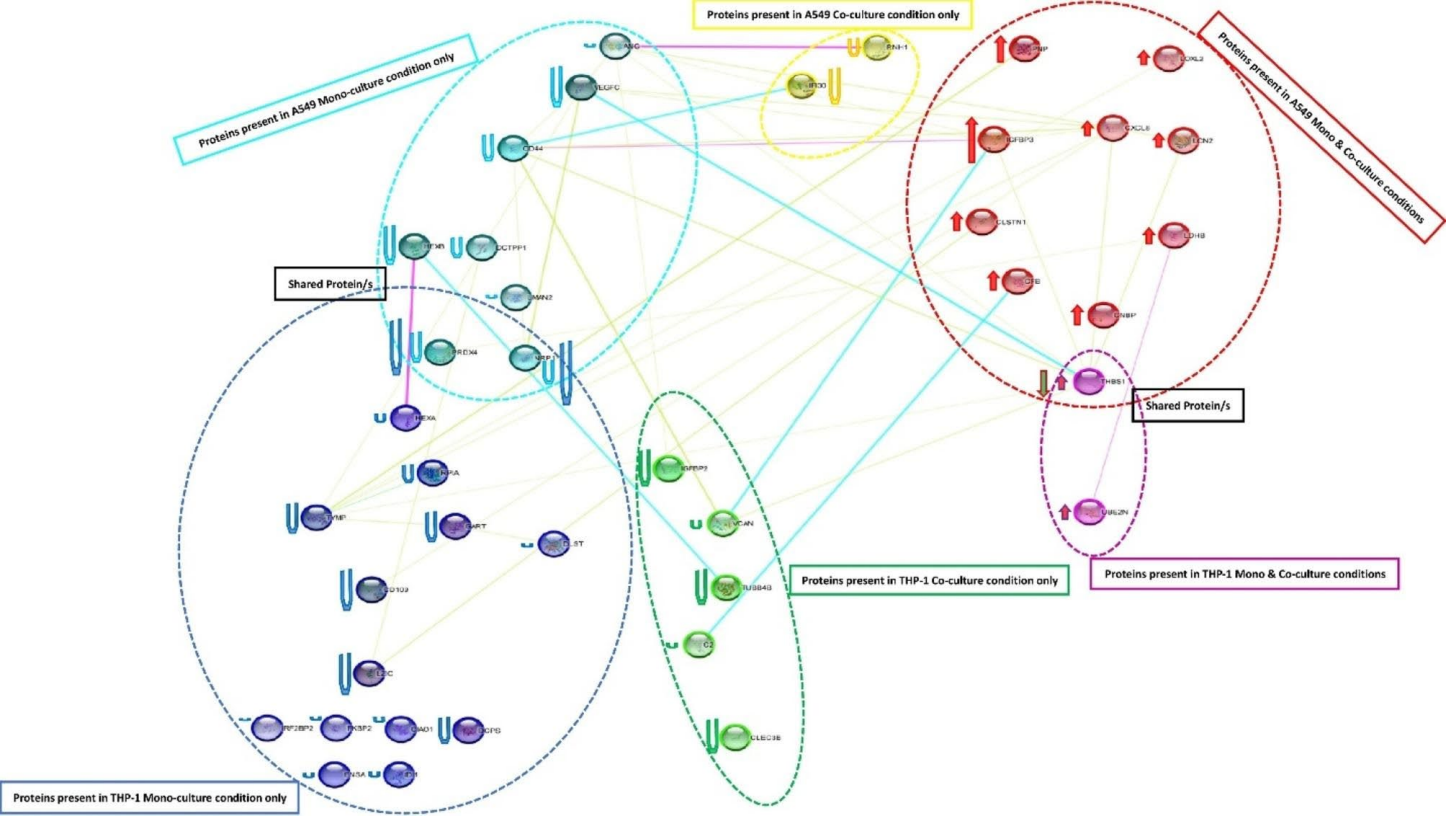
**Protein expression and interaction network analysis.** Protein interaction network generated with STRING v8.3 and visualized with Cytoscape v3.8.2 consisting of 39 proteins (nodes) connected by 40 protein-protein interactions (edges). There are six scenarios; in A549 mono-culture condition only 6 proteins are uniquely expressed (shown in turquoise) whereas in THP-1 mono-culture condition only 13 proteins are uniquely expressed (shown in blue). In these two scenarios 2 unique proteins are shared. In A549 co-culture condition only 2 proteins are uniquely expressed (shown in yellow) whereas in THP-1 co-culture condition only 5 proteins are uniquely expressed (shown in green). In A549 mono & co-culture condition, 9 proteins were upregulated with over 3 folds expression (shown in red). Whereas in THP-1 mono & co-culture condition, one protein was upregulated with over 3 folds expression (shown in purple). In these two scenarios a protein is shared, which is upregulated in THP-1 mono & co-culture condition while in A549 mono & co-culture condition, it is downregulated. In A549 mono & co-culture condition as well as THP-1 mono & co-culture condition, the size of the arrow depicts the fold expression, the more the fold-change the more the size and vice-versa. Whereas in rest of the four conditions, the size of U depicts the %CV, the more the %CV the more the size and vice-versa

To predict the interaction between selected secretory proteins and further understand their functional association, Cytoscape tool was employed. Cytoscape is a bioinformatics programme used to visualize molecular interaction networks and integrate them with gene expression data. The selected secretory proteins were imported from the string protein database into the Cytoscape tool to generate a protein–protein interaction map. String analysis of the mono-cultures of A549 as well as THP-1 and their respective co-culture scenarios (A549-Co CM, A549-Co/Mo CM, THP-1-Co CM and THP-1-Co/Mo CM) were collectively taken as a single entity. Overall, 6 proteins were found in A549 Mo-CM, 2 in A549 Co-CM, 9 in A549 Mo/Co-CM, 13 in THP-1 Mo-CM, 5 in THP-1 Co-CM and only one in THP-1 Mo/Co-CM. Apart from these secretory proteins, 2 proteins were shared between A549 Mo-CM as well as THP-1 Mo-CM scenarios and only one protein was shared between A549 Co/Mo CM as well as THP-1 Co/Mo CM scenarios. (Fig. 6).

To further get an insight into the pathways in which the identified proteins might be involved, BiNGO and Reacfoam based on voronoi visualization was carried out in Cytoscape. Pathway prediction was sorted by *p*-value, and only the most relevant pathways were selected. Protein(s) and their particular pathway(s) were established by comparing the number of proteins of a particular scenario, involved in a specific pathway(s) to the actual number of proteins involved in that specific pathway. For pathway analysis, all of the 6 identifiers in the A549 Mono-CM were detected in Reactome, with at least one of the proteins being involved in 180 pathways. In this scenario, the most significant pathways involved were related to IGF2BP pathway, Hyaluronan uptake and degradation, Hyaluronan metabolism and neutrophil degranulation pathway. In case of THP-1 Mono-CM, all 13 identifiers were found in Reactome, where 618 pathways were observed to be regulated by at least one of them. All the proteins in this group were significantly involved in most of the depicted pathways with FDR of each pathway being slightly towards the higher side, e.g., G2/M checkpoints, UCH proteinases, Regulation of Apoptosis, etc. However, in A549 Co-CM, only one out of 2 identifiers was observed in Reactome, with 34 pathways being modulated by it. The most significant pathways involved were related to INF-gamma pathway, tRNA-derived small RNA biogenesis and Interferon signaling. For THP-1 Co-CM, all 5 identifiers were found in Reactome, where 131 pathways were found to be regulated by at least one of them. All the pathways involved in this scenario were significant and FDR of every pathway was reasonable, e.g., Connexon trafficking, Connexon transport, Aggrephagy, etc. In the A549 Co/Mo CM scenario, 7 out of 9 identifiers were spotted in Reactome, where 232 pathways were hit by at least one of them. The most significant pathways involved were related to Interleukin-4 and Interleukin-13 signaling, Crosslinking of collagen fibrils and TP53 Regulating Transcription of Death Receptors and Ligands. 2 out of 2 identifiers in the THP1 Co/Mo CM sample were found in Reactome, where 139 pathways were observed to be governed by at least one of them. The most significant pathways involved were related to RUNX1-mediated regulation of megakaryocyte differentiation and platelet function, IRAK1 TLR7/8 or 9 stimulation recruiting IKK complex and intracellular fatty acid metabolism regulating insulin secretion. (Figure S4 and Supplementary Table 1).

**Fig. 7:**
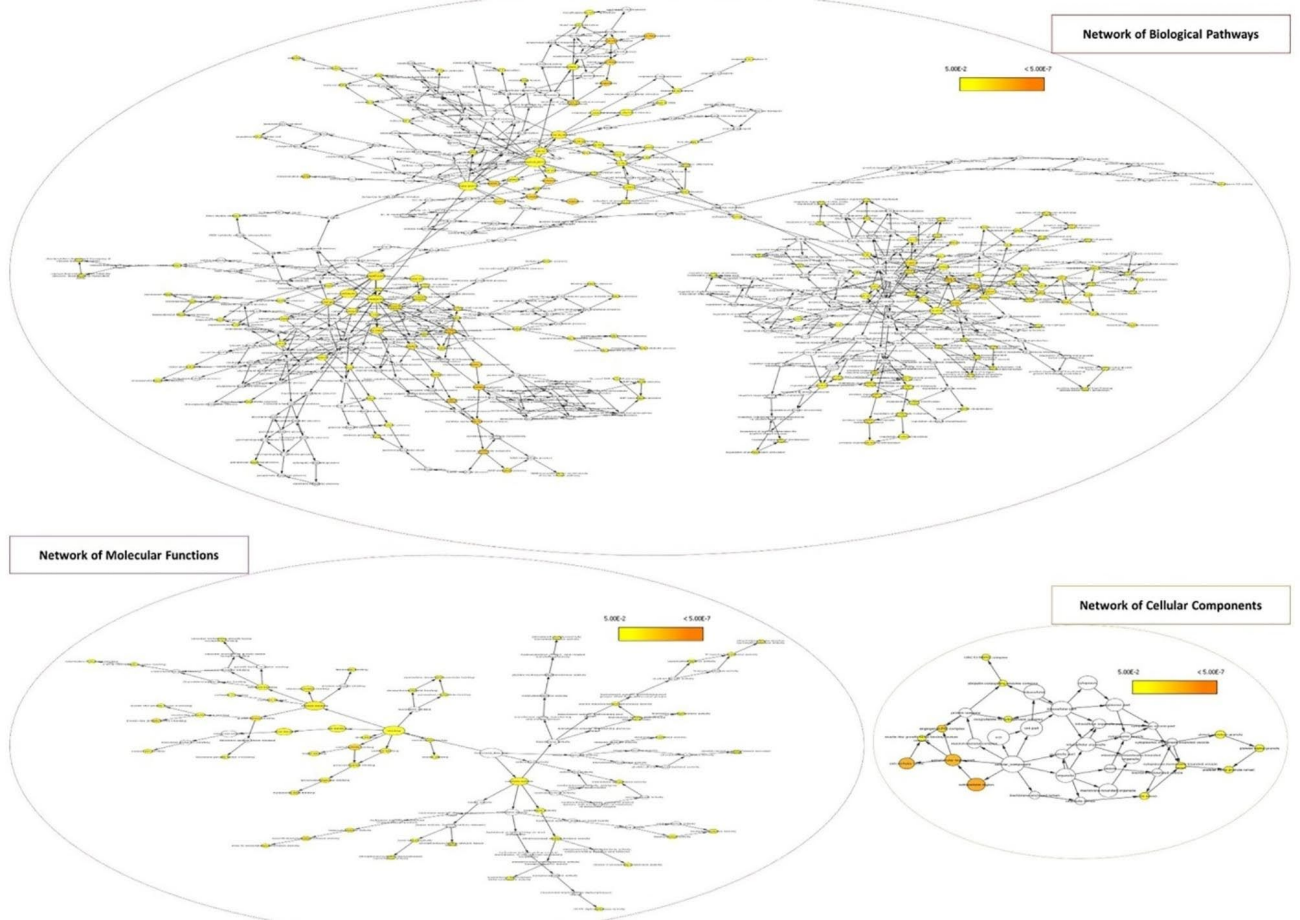
**Interactive tree constructed using BiNGO (A Biological Network Gene Ontology tool); generated overall biological pathways, molecular function and cellular component networks for proteins with significant fold-change and/or unique expression levels which qualified the %CV/fold-change and SignalP/SecretomeP scores in six scenarios combined.** In all the processes pooled together, 611 nodes with 961 edges were built by the proteins which qualified the %CV/fold-change and SignalP/SecretomeP scores in six scenarios combined. In GO-biological processes (demarcated in red), 326 biological processes with 533 overall biological connections were built. In GO-molecular functions (demarcated in purple), 105 molecular functions with 114 molecular/functional connections were built. In GO-cellular components (demarcated in orange), 34 cellular components with 56 cellular connections were built. The size of the node denotes the sum of proteins related to a GO term, whereas the color-code depicts the statistical significance (*p*-value) of the enrichment of a GO term

To generate an overall biological processes, cellular component and molecular function networks, the BiNGO (Biological Network Gene Ontology tool) was initially used to construct an interactive tree for the selected 39 proteins which qualified the < 10%CV/>3 fold-change from all the scenarios and for the seven proteins that were uniquely present in co-culture scenarios only (Proteins present in A549 and THP-1 co-culture scenarios only). Overall, in all the processes, 611 nodes with 961 edges were built by the proteins which qualified the < 10%CV/>3 fold-change scale in six scenarios combined. In GO-cellular components (demarcated in orange), 34 cellular components with 56 cellular connections were built. Of the 39 selected proteins, high number of proteins were involved in the biological processes of extracellular region (19/39), extracellular region part (13/39) and in extracellular space (12/39). When doing the same analysis for the seven proteins uniquely secreted in co-culture, 7 nodes were built with 8 edges. Out of these proteins, 6/7 (except Tubulin Beta-4B chain; TUBB3B) were involved in extra-cellular region, 5/7 (except gamma-interferon-inducible lysosomal thiol reductase preproprotein; IFI30 and TUBB3B) in the extracellular region part, 4/7 (except IFI30, TUBB3B and Ribonuclease Inhibitor isoform X1; RNH1) in extra-cellular space. Only RNH1 out of 7 was involved in angiogenin-PRI complex. Out of 4 cellular components, complement C2 isoform 1; C2, Tetranectin isoform 1; CLEC3B, Insulin-like growth factor-binding protein 2 isoform a; IGFBP2, Versican core protein isoform 1; VCAN and RNH1 were each involved in 3 of the four cellular components. Only IFI30 was involved in only one of the four cellular components. In GO-molecular functions (demarcated in purple), 105 molecular functions with 114 molecular/functional connections were built. Of the 39 shortlisted proteins, high number of proteins were involved in the protein binding (28/39), catalytic activity (21/39) and ion binding (16/39), however 7/39 of the proteins were each involved in carbohydrate, identical protein and receptor binding respectively. When doing the same analysis for the seven proteins that were uniquely present in co-culture scenarios only, 26 nodes were built with 28 edges. Out of these proteins, 2 out of 7 (CLEC3B and VCAN) were involved in sugar and carbohydrate binding, IGFBP2 insulin-like growth factor, I and II binding, RNH1 ribonuclease inhibitor and H activity as well as endoribonuclease activity producing 5’-phosphomonoesters respectively. Out of 9 molecular functions, IGFBP2, RNH1 and VCAN were each involved in 3 of the 9 molecular functions, however CLEC3B was involved in only two of the 9 molecular functions. In GO-biological processes (demarcated in red), the selected proteins were seen interacting with 326 nodes with 533 edges. Of the 39 shortlisted proteins, most were involved in metabolic process (24/39) followed by primary metabolic process (22/39), response to stimulus (18/39) and nitrogen compound metabolic process (13/39). 12 of the 39 proteins were involved in cellular nitrogen compound metabolic process as well as nucleic acid metabolic process. 11 of the 39 proteins were involved in both response to stress as well as small molecule metabolic process. 10/39 were associated with response to chemical stimulus. While 9/39 and 8/39 were involved in regulation of cell proliferation and immune system process as well as locomotion respectively. However, 7 of the 39 proteins were each involved in response to wounding, cellular catabolic process, blood vessel and vasculature development, cell migration, localization and motility of cell, nucleobase, nucleoside phosphate and nucleotide metabolic process, respectively. Surprisingly, in biological processes none of the seven proteins showed an association with any of the processes (Fig. 7).

### Confirmation of SILAC results by qRT-PCR

**Fig. 8:**
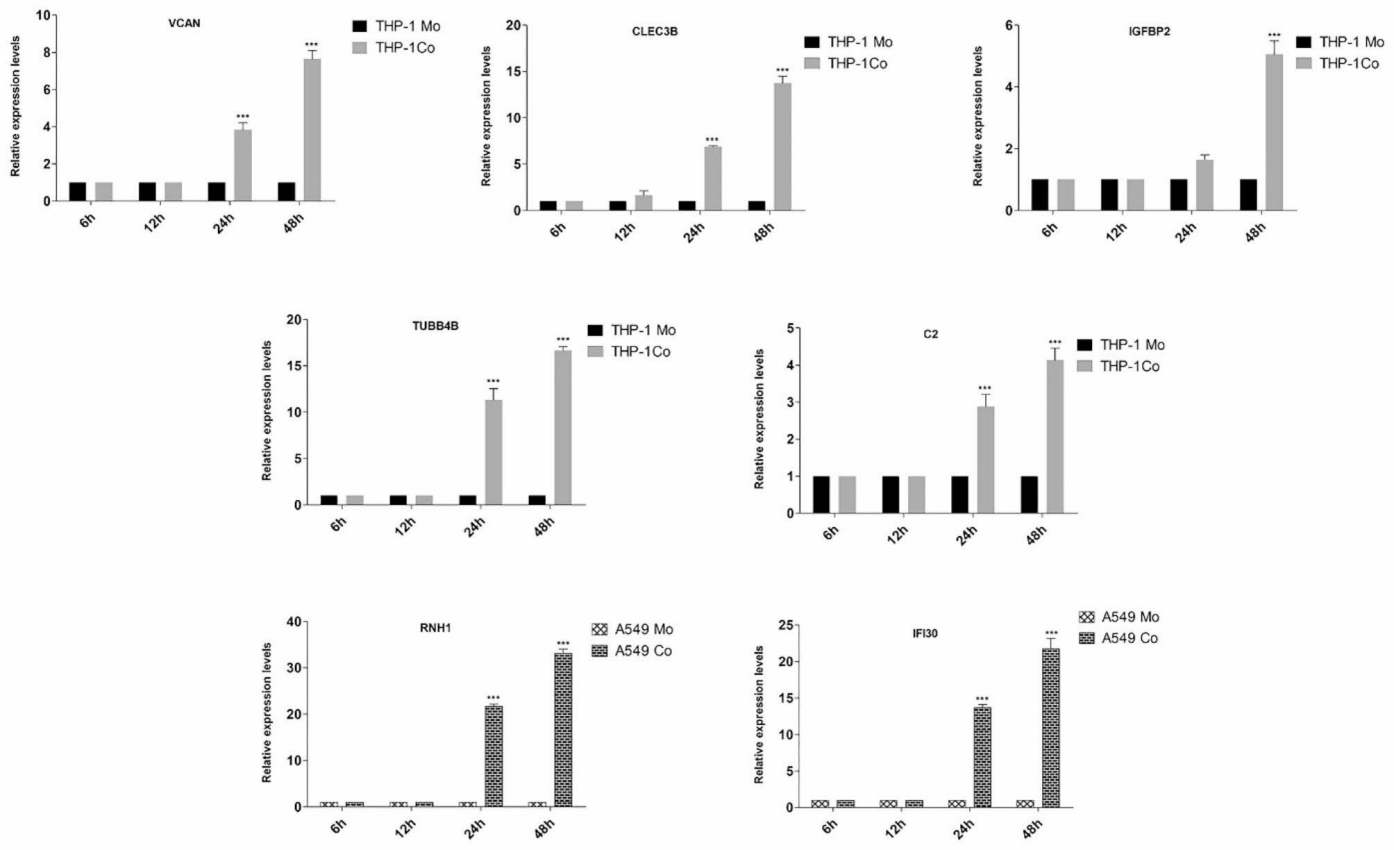
Quantitative mRNA expression levels of seven candidate proteins uniquely present in co-culture

In order to substantiate the SILAC results and to assess whether the co-culture of the two cell types affects the gene expression at transcriptomic level, the expression of seven candidate proteins uniquely present in co-culture was evaluated at the transcriptional level by qRT-PCR. Expression of each gene in a co-culture scenario was quantitated at 6 h, 12 h, 24 and 48 h time points respectively with reference to the respective monoculture. The results indicated that the mRNA expression of all the genes except IGFBP2 is induced after 24 h of co-culture (Fig. 8). The expression of IGFBP2 rises only after 48 h of co-culture. These findings are not only consistent with SILAC results but also provide an understanding that the release of such proteins might be due to their transcriptional upregulation.

### Correlation of the proteins uniquely secreted in co-culture with the hallmarks of cancer

**Fig. 9:**
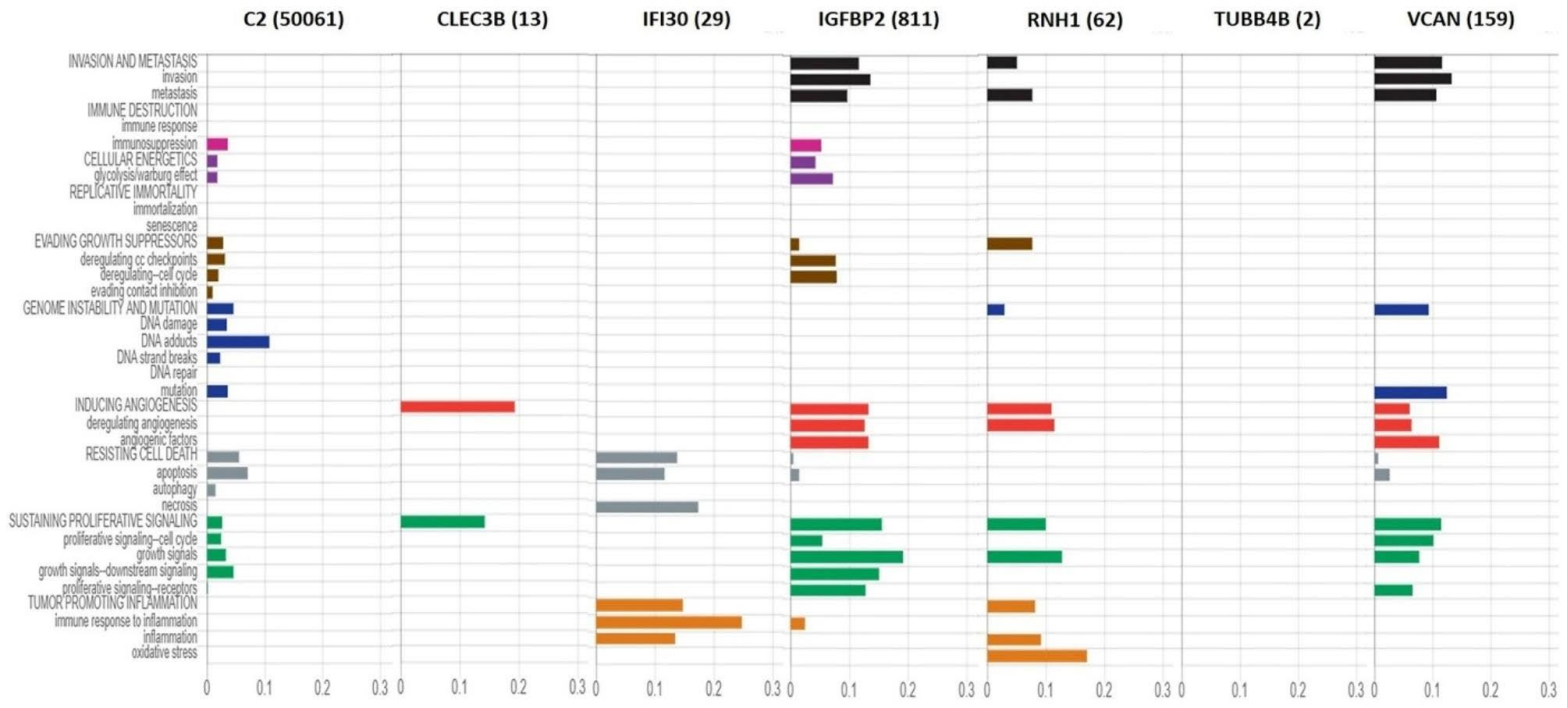
**Cancer Hallmarks Analytics Tool (CHAT) depicting the Hallmarks of Cancer associated with seven uniquely expressed proteins.** When analyzing all the proteins individually, one or many hallmarks were seen associated with them except the TUBB4B

Cancer Hallmarks Analytics Tool (CHAT) was employed to retrieve the Hallmarks of Cancer associated with the seven candidate proteins which were uniquely secreted in A549 and THP-1 co-cultures respectively. When analyzing proteins individually, C2 was not seen associated much to any of the hallmarks except in genome instability and mutation (DNA adducts). CLEC3B was seen majorly associated to two of the hallmarks, inducing angiogenesis and sustaining proliferative signaling. The hallmarks associated to IFI30 included resisting cell death (apoptosis and necrosis) and tumor promoting inflammation (inflammation and immune response associated to it) whereas those associated to IGFBP2 were invasion and metastasis, inducing angiogenesis (angiogenic deregulation and angiogenic factors) and sustaining proliferative signalling (growth signals, downstream signalling and receptor-based signalling). The hallmarks associated to RNH1 included angiogenesis (angiogenic deregulation), sustaining proliferative signalling (growth signals) and tumour promoting inflammation (oxidative stress). TUBB4B was not seen at all associated to any of the hallmarks of cancer, which could be due to the lack of hallmark association studies of this gene. The hallmarks associated to VCAN included invasion and metastasis, genome instability and mutation (mutation), inducing angiogenesis (angiogenic factors) and sustaining proliferative signaling (proliferative signaling). All of these analytics are depicted in the figure below (Fig. 9).

### Prognostic potential of candidate uniquely secreted proteins in non-small cell lung carcinoma

**Fig. 10:**
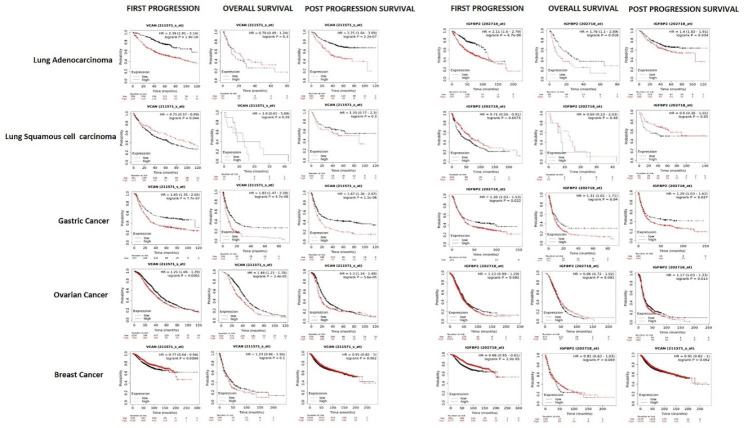
**Prognostic Potential of candidate proteins.** Kaplan–Meier plots depicting the association between the expression of Versican, IGFBP2 and the survival rate of patients with various tumours

The Kaplan–Meier plotter was extended to RNA-seq data databased in the TCGA, EGA, and GEO databases to analyze the association between the expression of seven candidate proteins uniquely present in co-culture and the survival rate of patients with various tumors. It was observed that the high expression of Versican was associated with an overall poor prognosis in Lung adenocarcinoma in comparison to Lung squamous cell carcinoma and various other solid tumors viz. gastric, ovarian and breast (Fig. 10). The parameters where Versican indicated poor prognosis included overall survival and first progression survival with Hazard ratio (HR) of 2.39 and 2.25 respectively. High expression of IGFBP2 was also found to correlate negatively with the overall survival (HR = 2.11), post progression survival (HR = 1.78) and first progression (HR = 1.4) (Fig. 10). All three parameters were high scoring in lung adenocarcinoma compared to Lung squamous cell carcinoma, gastric, ovarian and breast carcinomas. In a similar manner, expression of C2, IFI30 and TUBB4 was negatively associated with the disease outcome in lung adenocarcinoma compared to other carcinomas (Figure S5). However, for RNH1 and CLEC3B, contrasting observations were made and higher expression was found to affect the disease outcomes positively.

## Discussion

To evaluate the mechanisms by which lung cancer cells and monocytes modulate biological properties of each other in a reciprocal manner, we established an in vitro tumor microenvironment simulating model by co-culturing human model lung carcinoma cells, A549 and human monocytes, THP-1 in varying ratios. Expression of various cytokines commonly known to support tumor growth and metastatic dissemination was quantified to get an account of the interaction(s) occurring within the co-cultures [21–23]. All the tested co-cultures showed significant production of TNF-α, IL-6 and IL-10. However, the most effective response was observed at a co-culture ratio of 1:10, thus pointing at the abundance of monocyte activator(s) in the co-culture milieu in this ratio. The levels of the prototypical pro-inflammatory cytokines, TNF-α and IL-6 were observed to increase with time, with response peaking at 48 h time point, however, IL-10, an anti-inflammatory cytokine, showed an optimal response at 48 h. Early release of TNF-α and IL-6 as well as the expression of IL-10 at the highest time point hints at the differentiation of monocytes in co-culture towards a phenotype that resembles M2 macrophages as compared to the M1 phenotype observed at earlier time points. Both TNF-α and IL-6 have been shown to be classical indicators of macrophage activation [9] and in vivo demonstrated to mediate tumor promotion in various human cancers [24, 25]. In our settings, accentuated migration, invasion, colony formation as well as transition of A549 cells to mesenchymal state when cultured in 1:10 co-culture conditioned medium can be attributed to such characteristics of these cytokines.

Taking the above findings into consideration, we further sought to identify secretory proteins that mediate the interactions between lung carcinoma cells and monocytes in co-culture by using SILAC based proteomic approach. We applied the ‘double labeled’ SILAC approach to investigate protein secretion and regulation during co-culture of lung carcinoma cell and monocytes. The advantage of the SILAC-based experimental strategy is that the proteins secreted by each cell type in co-culture can be distinguished and identified by the stable isotope amino acids that had been incorporated [26, 27]. We identified a total of 414 potentially secreted proteins which were subjected to various filters of %CV, fold-change and neural-network probability algorithmic tools, SecretomeP and SignalP to select authentic secretory proteins across all the scenarios. Overall, 39 proteins were shortlisted among which 20 were classical secretory proteins (CSPs) while 19 were non-classical secretory proteins (NCSPs). Which were finally were analyzed through STRING protein database and Reactome knowledgebase system respectively to investigate the intrinsic interactions exhibited by them and to analyze the pathways in which these proteins might be involved [28–30]. Through the analysis of biological processes, cellular component and molecular function using BINGO, it was revealed that the 39 selected proteins are mainly localized in extracellular region and are involved in protein binding, catalytic activity and ion binding, identical protein and receptor binding. The molecular functions attributed to these proteins primarily included metabolic process, response to stimulus and stress.

Among the 39 selected secretory proteins we focused only on the 7 unique proteins in the co-culture scenarios for downstream analysis because of their peculiar secretion in these scenarios. These included RNH1 and IFI30 in A549 Co-CM and CLEC3B, VCAN, IGFBP2, C2, TUBB4B in THP-1-Co CM scenarios. Validation of the gene expression of these protein candidates by qRT-PCR analysis depicted a general consonance between the proteins identified and their expression at transcriptional level. In addition, qRT-PCR revealed that induction in the gene expression of all the candidate proteins occurs after 24 h of co-culture except IGFBP2 whose expression was seen to rise only after 48 h of co-culture. The finding provides an understanding that the release of these proteins occurs due to their transcriptional upregulation. In addition, we demonstrated the proteins uniquely secreted in A549 and THP-1 co-cultures except TUBB4 are associated with one or the other hallmark of cancer which supports their candidature as the proteins involved in lung cancer pathogenesis. This candidature was further evidenced by evaluating their prognostic potential using the related RNA-seq data available in the TCGA, EGA, and GEO databases. The high expression of the VCAN, IGFBP2, C2, and TUBB4 (proteins uniquely secreted in THP-1 co-culture) and IFI30 (proteins uniquely secreted in A549 co-culture) were associated with poor prognosis and correlated negatively with disease outcomes. Intriguingly such features of these proteins were specific to lung adenocarcinoma compared to lung squamous cell carcinoma, gastric, ovarian and breast carcinomas.

The secretion of versican (VCAN) by monocytes due to their interaction with lung carcinoma cells is being reported in this study for the first time. Versican is an extracellular aggregating proteoglycan involved in the assembly of the extracellular matrix [31]. Versican inhibits cancer cell attachment to stromal matrix, thereby facilitating cancer cell migration and invasion [32, 33]. Ligation of TLR2 by versican has been reported to activate stromal cell populations in tumor microenvironment and subsequent inflammatory cytokine secretion [34]. In our study, the augmented migration, invasion and EMT of A549 cells, when grown in the co-culture conditioned medium, can therefore be related to the action of THP-1 secreted versican in the co-culture conditioned medium. Conversely, versican secreted by THP-1 cells can ligate TLR-2 on these cells to induce the production of inflammatory cytokines in an autocrine fashion which generates an inflammatory microenvironment congenial for tumor progression [9]. Versican expression has been correlated with poor prognosis, increased TAM infiltration, poor tumor differentiation, and a higher tumor-grade metastasis (TNM stage) in a variety of cancers [35]. Therefore our findings are in line with these studies and lay further impetus on the importance of Versican in lung cancer progression.

Tetranectin (CLEC3B) is a lectin with specific binding affinity for plasminogen. This binding of Tetranectin to plasminogen leads to the activation of plasminogen-cascade that triggers the proteolytic processing and degradation of the extracellular matrix and thereby cancer cell migration and invasion [36]. In our experimental setup, prometastatic effects of the co-culture medium can be ascribed to the presence of Tetranectin in the co-culture medium secreted by monocytes due to their interaction with lung carcinoma cells. The results further provide clue that in lung cancers, presence of monocytes in tumor microenvironment triggers the release of Tetranectin from cancer cells, thereby promoting cancer progression.

Insulin like growth factor binding protein 2 (IGFBP2) is the protein secreted by THP-1 cells in co-culture with A549 cells. IGFBP2 promotes tumor cellular proliferation, migration, invasion, angiogenesis, epithelialtomesenchymal transition and is highly elevated in serum or tissue in patients with malignant tumors. It has been found to be overexpressed in a broad spectrum of tumors including lung cancer wherein higher plasma levels of IGFBP2 have been positively associated with tumor size, lymph node metastasis, advanced tumor stage, and shorter overall survival [37]. In our co-culture medium set-up, observed metastatic effects could be related to the secretion of IGFBP2 by monocytes. Overall the finding provide an insight into the role played by IGFBP2 in lung cancer metastasis.

TUBB4B is an isotype of β-tubulin, a member of the tubulin family that forms the building blocks of the microtubule networks. This protein is reported to be overexpressed in glioblastoma, ovarian and prostate cancers [38–40], although its downregulation has been shown to initiate EMT in colon cancer [41]. A recent study has implicated TUBB4B as an essential factor required for the maintenance of cancer stem cell niche via its interaction with Ephrin-B1. In the context of our study, we assume that the TUBB4B secreted by THP-1 cells during co-culture might interact with Ephrin-B1 on A549 cells which in turn by ligating ephrin receptor might promote cell migration and invasion [42, 43]. Thus the finding provides evidence that lung cancer cells modulate monocytes to release TUBB4B which in turn promotes cancer metastasis.

C2, a complement protein was secreted by THP-1 cells in co-culture with A549 cells. In solid tumors both cancer and stromal cells have the ability to produce complement proteins. Amplification of the complement system has been shown to promote proliferation, migration, and epithelial-mesenchymal transition [44, 45]. Therefore it can be argued that the presence of C2 in the co-culture conditioned medium supports the metastasis properties of A549 cells.

Interferon-gamma-inducible protein 30 (IFI30) was secreted by A549 cells in co-culture with THP-1. Dysregulated expression of IFI30 has been associated with human cancers. IFI30 is upregulated in breast cancer and melanoma and has been linked to disease progression in prostate cancer [46]. Gene Ontology (GO) analysis shows that IFI30 is associated with enhanced leukocyte-mediated immune and inflammatory responses. In addition, studies on the tumor microenvironment show that increased infiltration of M2-type macrophages was associated with high IFI30 expression [47]. Our results in line with these findings suggest that lung cancer cell secreted IFI30 promotes inflammatory response observed in co-culture scenario.

## Conclusion

In conclusion, this study identifies IFI30, RNH1 and CLEC3B, VCAN, IGFBP2, C2, TUBB4B as the proteins secreted by lung carcinoma cells and monocytes respectively as a result of their mutual interaction. These proteins may either act in an autocrine/paracrine manner by ligating cell surface receptors and eventually support metastasis. These proteins may thus be considered as attractive targets for therapeutic targeting to curb the lung cancer progression.

### Electronic supplementary material

Below is the link to the electronic supplementary material.

Supplementary Material 1

Supplementary Material 2

Supplementary Material 3

Supplementary Material 4

Supplementary Material 5

Supplementary Material 6

Supplementary Material 7

Supplementary Material 8

## Data Availability

The mass spectrometry proteomics data have been deposited to the ProteomeXchange Consortium via the PRIDE partner repository with the dataset identifier PXD040866. The data can be accessed at http://www.ebi.ac.uk/pride/archive/projects/PXD040866. Rest of the data needed to evaluate the conclusions in the study are present in the manuscript and the supplementary materials. Any additional data required will be made available upon reasonable request to the corresponding author.

## Abbreviations

TNF-𝛼: Tumor Necrosis Factor-Alpha
IL-6: Interleukin-6
IL-10: Interleukin-10
SILAC: Stable Isotope Labeling by Amino Acids in Cell Culture
LC-MS/MS: Liquid Chromatography with tandem mass spectrometry
CHAT: The Cancer Hallmarks Analytics Tool
EMT: Epithelial Mesenchymal Transition
TIMs: Tumor Infiltrating Macrophages
TAMs: Tumor Associated Macrophages
CLEC3B: Tetranectin
VCAN: Versican
IGFBP2: Insulin-like Growth Factor-2
RNH1: Ribonuclease Inhibitor isoform X1
C2: Complement C2 Isoform 1
TUBB4: Tubulin Beta 4B Class IVB
TCGA: The Cancer genome Atlas
EGA: European Genome-phenome Archive
GEO: Gene Expression Omnibus
BiNGO: Biological Networks Gene Ontology
