# Supplementary figures and images for: Dissection of paracrine/autocrine interplay in lung tumor microenvironment mimicking cancer cell-monocyte co-culture models reveals proteins that promote inflammation and metastasis
Source: BMC Cancer. 2023 Oct 2;23:926. doi: 10.1186/s12885-023-11428-7 (PMC10544320; doi:10.1186/s12885-023-11428-7)

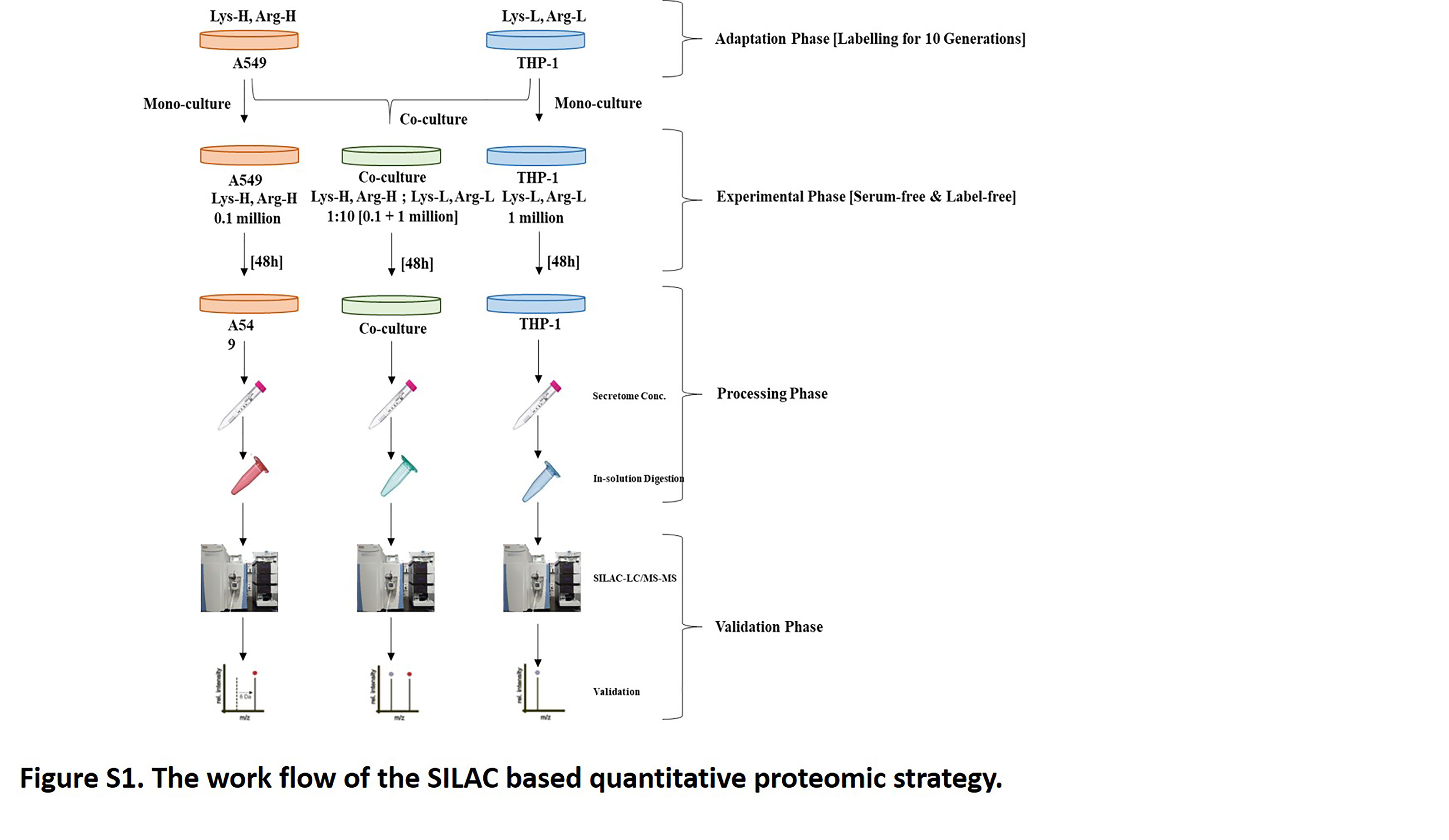

Supplement: Supplementary file 1 — Supplementary Material 1 [file 12885_2023_11428_MOESM1_ESM.jpg]

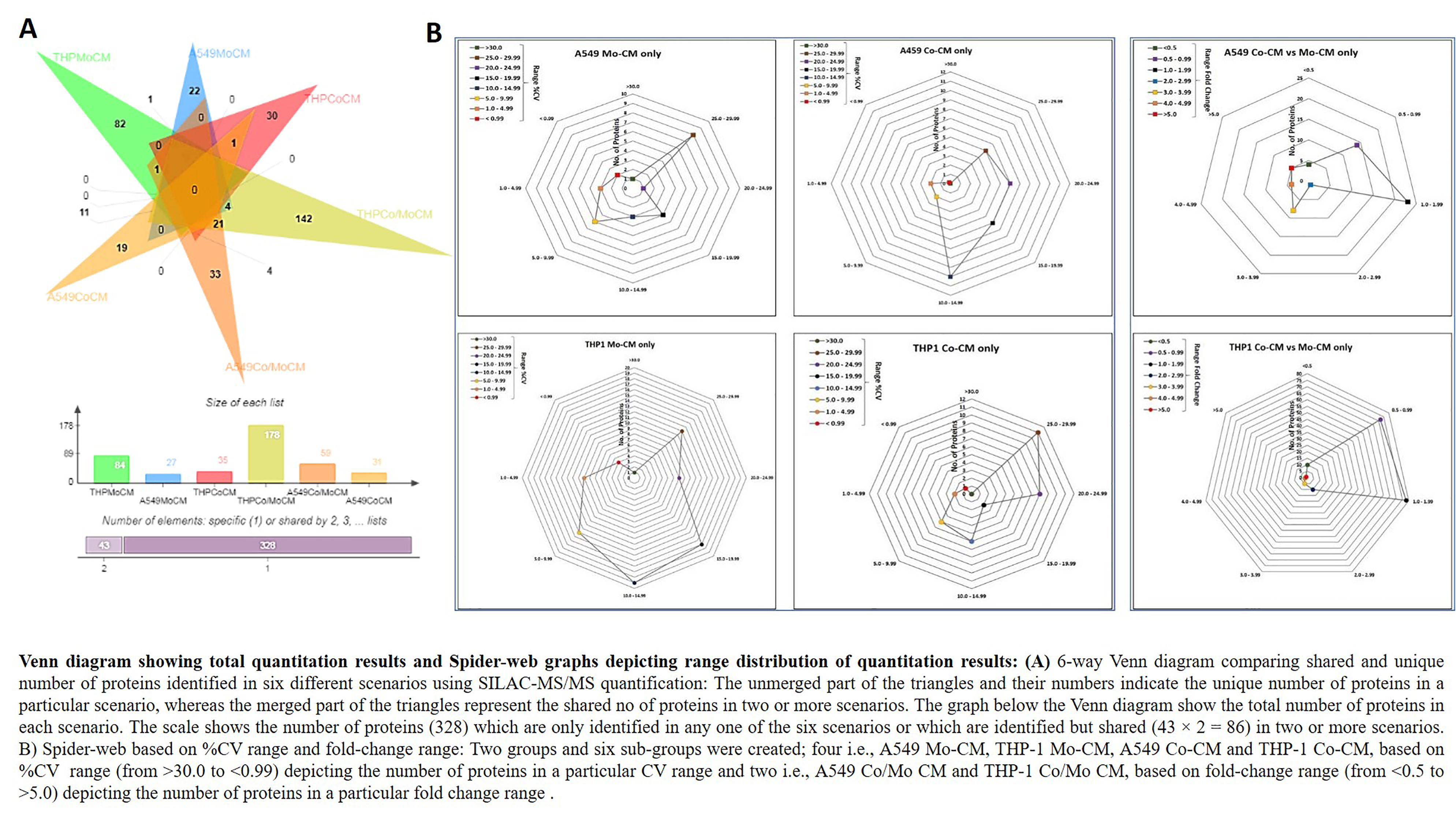

Supplement: Supplementary file 2 — Supplementary Material 2 [file 12885_2023_11428_MOESM2_ESM.jpg]

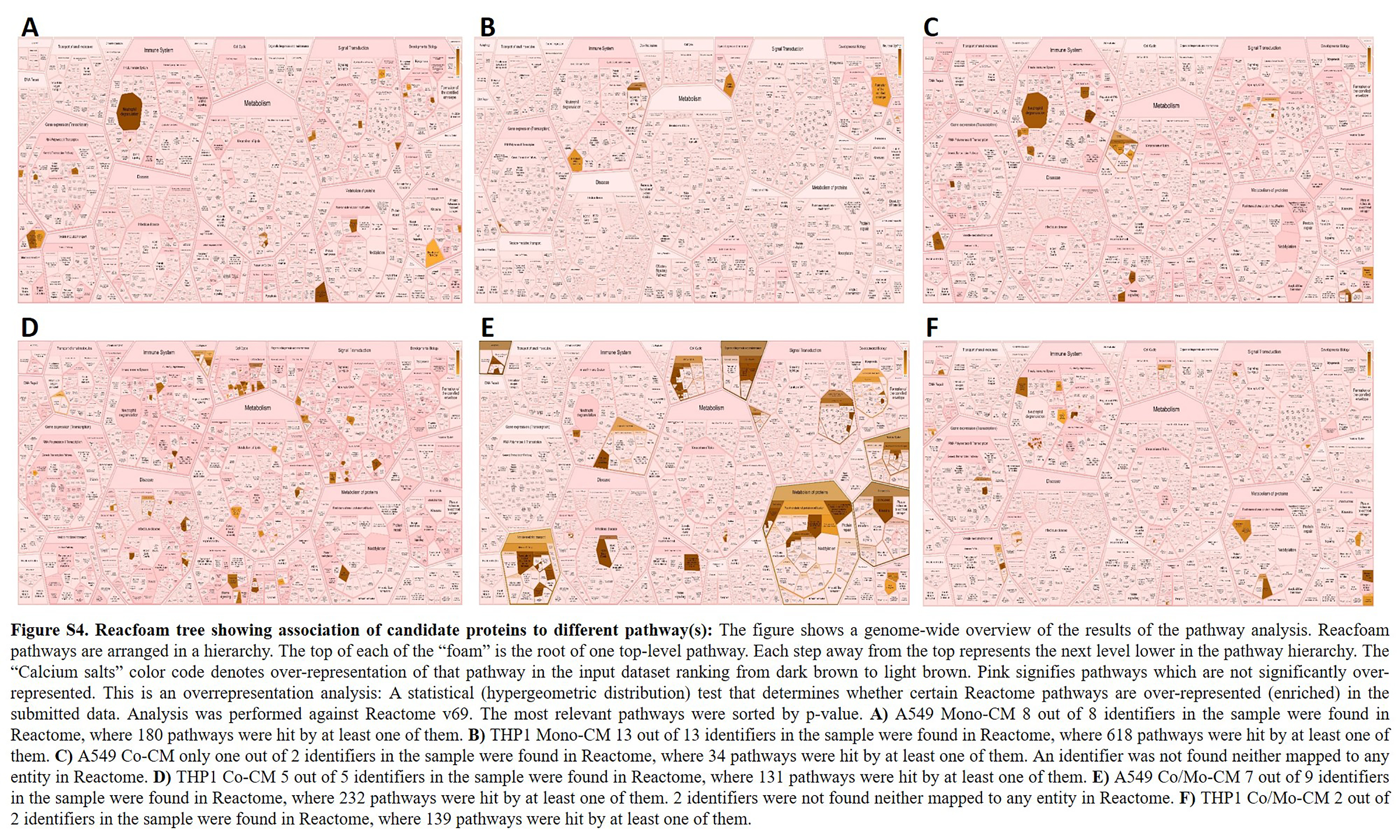

Supplement: Supplementary file 3 — Supplementary Material 3 [file 12885_2023_11428_MOESM3_ESM.jpg]

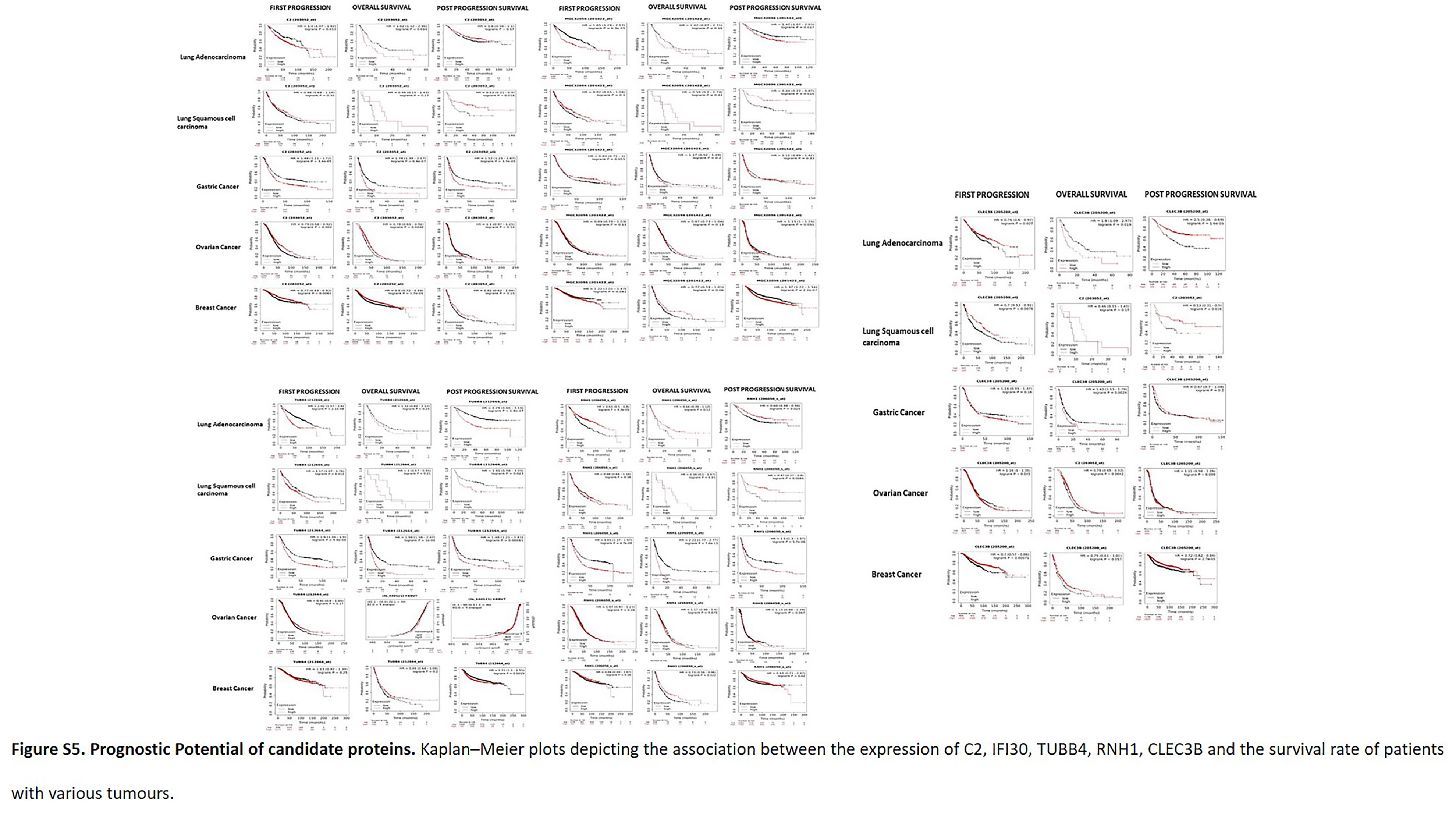

Supplement: Supplementary file 4 — Supplementary Material 4 [file 12885_2023_11428_MOESM4_ESM.jpg]

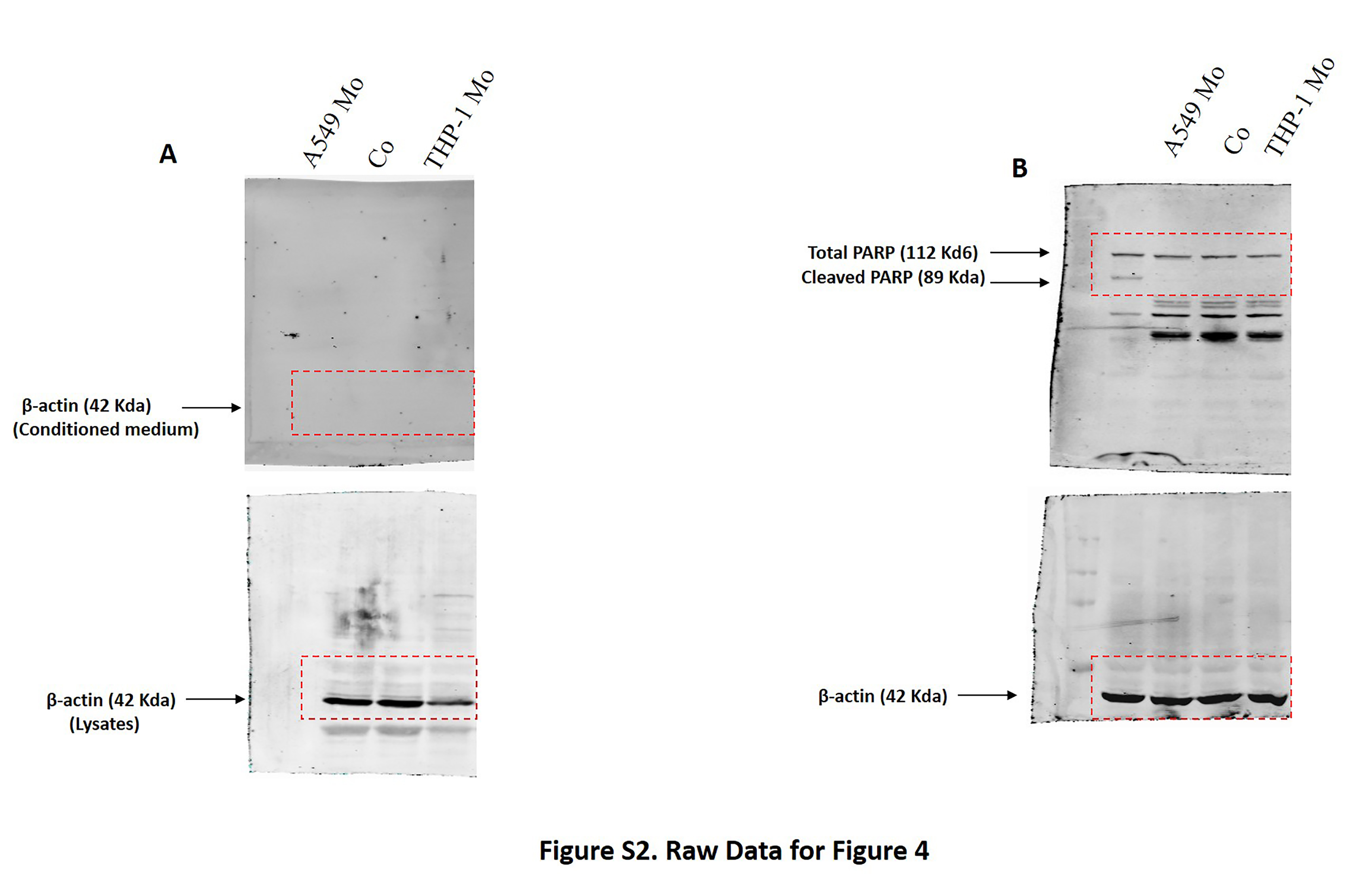

Supplement: Supplementary file 8 — Supplementary Material 8 [file 12885_2023_11428_MOESM8_ESM.jpg]
