# Supplementary material for: Dissection of paracrine/autocrine interplay in lung tumor microenvironment mimicking cancer cell-monocyte co-culture models reveals proteins that promote inflammation and metastasis
Source: BMC Cancer. 2023 Oct 2;23:926. doi: 10.1186/s12885-023-11428-7 (PMC10544320; doi:10.1186/s12885-023-11428-7)
Supplement: Supplementary file 5 — Supplementary Material 5 [file 12885_2023_11428_MOESM5_ESM.docx]

**Supplementary Table1.** Most significant pathways sorted by p-value from the select proteins in different scenarios.

The following table shows the 25 most significant pathways sorted by p-value from the select proteins in A549 Mo-CM:

| Pathway name | Entities | | | | | Reactions | |
| --- | --- | --- | --- | --- | --- | --- | --- |
|  | **found** | **ratio** | **p-value** | **FDR*** | **found** | | **ratio** |
| **Insulin-like Growth Factor-2 mRNA Binding Proteins (IGF2BPs/IMPs/VICKZs) bind RNA** | 5 / 23 | 0.001 | 2.18e-11 | 3.99e-09 | 2 / 3 | | 2.21e-04 |
| **Hyaluronan uptake and degradation** | 2 / 35 | 0.002 | 4.97e-04 | 0.039 | 5 / 10 | | 7.37e-04 |
| **Hyaluronan metabolism** | 2 / 40 | 0.002 | 6.47e-04 | 0.039 | 5 / 13 | | 9.58e-04 |
| **Neutrophil degranulation** | 4 / 480 | 0.022 | 9.15e-04 | 0.041 | 4 / 10 | | 7.37e-04 |
| **Defective HEXB causes GM2G2** | 1 / 4 | 1.87e-04 | 0.004 | 0.134 | 3 / 3 | | 2.21e-04 |
| **Defective HEXA causes GM2G1** | 1 / 5 | 2.33e-04 | 0.005 | 0.14 | 3 / 3 | | 2.21e-04 |
| **Transfer of LPS from LBP carrier to CD14** | 1 / 6 | 2.80e-04 | 0.006 | 0.145 | 2 / 2 | | 1.47e-04 |
| **Attachment of GPI anchor to uPAR** | 1 / 11 | 5.13e-04 | 0.01 | 0.204 | 2 / 2 | | 1.47e-04 |
| **NrCAM interactions** | 1 / 11 | 5.13e-04 | 0.01 | 0.204 | 1 / 4 | | 2.95e-04 |
| **Interaction with Cumulus Cells and the Zona Pellucida** | 1 / 14 | 6.53e-04 | 0.013 | 0.208 | 1 / 2 | | 1.47e-04 |
| **Antagonism of Activin by Follistatin** | 1 / 14 | 6.53e-04 | 0.013 | 0.208 | 1 / 2 | | 1.47e-04 |
| **Prostanoid ligand receptors** | 1 / 16 | 7.47e-04 | 0.015 | 0.221 | 1 / 8 | | 5.89e-04 |
| **Glycosaminoglycan metabolism** | 2 / 207 | 0.01 | 0.016 | 0.221 | 9 / 88 | | 0.006 |
| **RUNX3 Regulates Immune Response and Cell Migration** | 1 / 19 | 8.87e-04 | 0.018 | 0.222 | 1 / 5 | | 3.68e-04 |
| **RUNX2 regulates genes involved in cell migration** | 1 / 20 | 9.33e-04 | 0.019 | 0.222 | 1 / 7 | | 5.16e-04 |
| **Keratan sulfate degradation** | 1 / 22 | 0.001 | 0.02 | 0.224 | 2 / 7 | | 5.16e-04 |
| **VEGF ligand-receptor interactions** | 2 / 28 | 0.001 | 0.026 | 0.225 | 3 / 4 | | 2.95e-04 |
| **VEGF binds to VEGFR leading to receptor dimerization** | 2 / 28 | 0.001 | 0.026 | 0.225 | 2 / 3 | | 2.21e-04 |
| **CS/DS degradation** | 1 / 29 | 0.001 | 0.027 | 0.225 | 2 / 10 | | 7.37e-04 |
| **Neurophilin interactions with VEGF and VEGFR** | 3 / 30 | 0.001 | 0.028 | 0.225 | 2 / 2 | | 1.47e-04 |
| **SEMA3A-Plexin repulsion signalling by inhibiting Integrin adhesion** | 1 / 32 | 0.001 | 0.029 | 0.225 | 6 / 8 | | 5.89e-04 |
| **tRNA-derived small RNA (tsRNA or tRNA-related fragment, tRF) biogenesis** | 1 / 33 | 0.002 | 0.03 | 0.225 | 1 / 4 | | 2.95e-04 |
| **Thromboxane signalling through TP receptor** | 1 / 35 | 0.002 | 0.032 | 0.225 | 3 / 8 | | 5.89e-04 |
| **Sema3A PAK dependent Axon repulsion** | 1 / 45 | 0.002 | 0.041 | 0.277 | 3 / 6 | | 4.42e-04 |
| **Acrosome Reaction and Sperm:Oocyte Membrane Binding** | 1 / 45 | 0.002 | 0.041 | 0.277 | 1 / 3 | | 2.21e-04 |

The following table shows the 25 most significant pathways sorted by p-value from the select proteins in THP-1 Mo-CM:

| Pathway name | Entities | | | | Reactions | |
| --- | --- | --- | --- | --- | --- | --- |
|  | **found** | **ratio** | **p-value** | **FDR*** | **found** | **ratio** |
| **Cross-presentation of soluble exogenous antigens (endosomes)** | 2 / 53 | 0.002 | 0.004 | 0.267 | 1 / 6 | 4.42e-04 |
| **SCF-beta-TrCP mediated degradation of Emi1** | 2 / 55 | 0.003 | 0.004 | 0.267 | 1 / 3 | 2.21e-04 |
| **Regulation of ornithine decarboxylase (ODC)** | 2 / 57 | 0.003 | 0.004 | 0.267 | 1 / 4 | 2.95e-04 |
| **Vif-mediated degradation of APOBEC3G** | 2 / 57 | 0.003 | 0.004 | 0.267 | 1 / 4 | 2.95e-04 |
| **G2/M Checkpoints** | 3 / 194 | 0.009 | 0.005 | 0.267 | 3 / 24 | 0.002 |
| **UCH proteinases** | 3 / 202 | 0.009 | 0.005 | 0.267 | 8 / 11 | 8.10e-04 |
| **Evasion of Oncogene Induced Senescence Due to Defective p16INK4A binding to CDK4 and CDK6** | 1 / 3 | 1.40e-04 | 0.005 | 0.267 | 1 / 1 | 7.37e-05 |
| **Evasion of Oxidative Stress Induced Senescence Due to Defective p16INK4A binding to CDK4 and CDK6** | 1 / 3 | 1.40e-04 | 0.005 | 0.267 | 1 / 1 | 7.37e-05 |
| **Autodegradation of Cdh1 by Cdh1:APC/C** | 2 / 64 | 0.003 | 0.006 | 0.267 | 1 / 2 | 1.47e-04 |
| **Hh mutants are degraded by ERAD** | 2 / 64 | 0.003 | 0.006 | 0.267 | 1 / 6 | 4.42e-04 |
| **CDT1 association with the CDC6:ORC:origin complex** | 2 / 66 | 0.003 | 0.006 | 0.267 | 1 / 3 | 2.21e-04 |
| **Hh mutants abrogate ligand secretion** | 2 / 68 | 0.003 | 0.006 | 0.267 | 1 / 7 | 5.16e-04 |
| **Degradation of AXIN** | 2 / 71 | 0.003 | 0.007 | 0.267 | 2 / 8 | 5.89e-04 |
| **APC/C:Cdc20 mediated degradation of Securin** | 2 / 72 | 0.003 | 0.007 | 0.267 | 1 / 3 | 2.21e-04 |
| **Regulation of RAS by GAPs** | 2 / 72 | 0.003 | 0.007 | 0.267 | 1 / 5 | 3.68e-04 |
| **Regulation of activated PAK-2p34 by proteasome mediated degradation** | 2 / 74 | 0.003 | 0.007 | 0.267 | 1 / 2 | 1.47e-04 |
| **Orc1 removal from chromatin** | 2 / 77 | 0.004 | 0.008 | 0.267 | 1 / 4 | 2.95e-04 |
| **CDK-mediated phosphorylation and removal of Cdc6** | 2 / 80 | 0.004 | 0.009 | 0.267 | 1 / 4 | 2.95e-04 |
| **Defective HEXA causes GM2G1** | 1 / 5 | 2.33e-04 | 0.009 | 0.267 | 3 / 3 | 2.21e-04 |
| **Regulation of Apoptosis** | 2 / 85 | 0.004 | 0.01 | 0.278 | 1 / 5 | 3.68e-04 |
| **Autodegradation of the E3 ubiquitin ligase COP1** | 2 / 86 | 0.004 | 0.01 | 0.278 | 1 / 5 | 3.68e-04 |
| **p53-Independent DNA Damage Response** | 3 / 92 | 0.004 | 0.011 | 0.278 | 4 / 4 | 2.95e-04 |
| **Ubiquitin Mediated Degradation of Phosphorylated Cdc25A** | 3 / 92 | 0.004 | 0.011 | 0.278 | 4 / 4 | 2.95e-04 |
| **p53-Independent G1/S DNA damage checkpoint** | 3 / 92 | 0.004 | 0.011 | 0.278 | 4 / 4 | 2.95e-04 |
| **Signaling by membrane-tethered fusions of PDGFRA or PDGFRB** | 1 / 7 | 3.27e-04 | 0.012 | 0.288 | 2 / 2 | 1.47e-04 |

The following table shows the 25 most significant pathways sorted by p-value from the select proteins in A549 Co-CM:

| Pathway name | Entities | | | | | Reactions | |
| --- | --- | --- | --- | --- | --- | --- | --- |
|  | **found** | **ratio** | **p-value** | **FDR*** | **found** | | **ratio** |
| **Interferon gamma signaling** | 2 / 468 | 0.022 | 0.003 | 0.094 | 2 / 16 | | 0.001 |
| **tRNA-derived small RNA (tsRNA or tRNA-related fragment, tRF) biogenesis** | 1 / 33 | 0.002 | 0.006 | 0.104 | 1 / 4 | | 2.95e-04 |
| **Interferon Signaling** | 2 / 1,034 | 0.048 | 0.013 | 0.11 | 2 / 69 | | 0.005 |
| **Adherens junctions interactions** | 1 / 137 | 0.006 | 0.025 | 0.11 | 1 / 16 | | 0.001 |
| **Cristae formation** | 1 / 168 | 0.008 | 0.031 | 0.11 | 1 / 2 | | 1.47e-04 |
| **Gene Silencing by RNA** | 1 / 174 | 0.008 | 0.032 | 0.11 | 1 / 40 | | 0.003 |
| **MHC class II antigen presentation** | 1 / 203 | 0.009 | 0.037 | 0.11 | 1 / 26 | | 0.002 |
| **Cell-cell junction organization** | 1 / 215 | 0.01 | 0.04 | 0.11 | 1 / 21 | | 0.002 |
| **Formation of the cornified envelope** | 1 / 227 | 0.011 | 0.042 | 0.11 | 1 / 27 | | 0.002 |
| **Keratinization** | 1 / 312 | 0.015 | 0.057 | 0.11 | 1 / 34 | | 0.003 |
| **Neurofascin interactions** | 1 / 321 | 0.015 | 0.059 | 0.11 | 1 / 7 | | 5.16e-04 |
| **Regulation of necroptotic cell death** | 1 / 414 | 0.019 | 0.075 | 0.11 | 1 / 18 | | 0.001 |
| **Cell junction organization** | 1 / 423 | 0.02 | 0.077 | 0.11 | 1 / 37 | | 0.003 |
| **RIPK1-mediated regulated necrosis** | 1 / 445 | 0.021 | 0.081 | 0.11 | 1 / 34 | | 0.003 |
| **Ephrin signaling** | 1 / 492 | 0.023 | 0.089 | 0.11 | 1 / 11 | | 8.10e-04 |
| **Mitochondrial biogenesis** | 1 / 505 | 0.024 | 0.091 | 0.11 | 1 / 36 | | 0.003 |
| **Regulated Necrosis** | 1 / 517 | 0.024 | 0.093 | 0.11 | 1 / 57 | | 0.004 |
| **Cell-Cell communication** | 1 / 617 | 0.029 | 0.11 | 0.11 | 1 / 60 | | 0.004 |
| **Cytokine Signaling in Immune system** | 2 / 3,593 | 0.168 | 0.133 | 0.133 | 2 / 708 | | 0.052 |
| **EPH-Ephrin signaling** | 1 / 758 | 0.035 | 0.134 | 0.134 | 1 / 56 | | 0.004 |
| **L1CAM interactions** | 1 / 893 | 0.042 | 0.157 | 0.157 | 1 / 54 | | 0.004 |
| **XBP1(S) activates chaperone genes** | 1 / 1,006 | 0.047 | 0.175 | 0.175 | 1 / 47 | | 0.003 |
| **IRE1alpha activates chaperones** | 1 / 1,013 | 0.047 | 0.176 | 0.176 | 1 / 53 | | 0.004 |
| **Organelle biogenesis and maintenance** | 1 / 1,029 | 0.048 | 0.179 | 0.179 | 1 / 86 | | 0.006 |
| **Unfolded Protein Response (UPR)** | 1 / 1,491 | 0.07 | 0.251 | 0.251 | 1 / 94 | | 0.007 |

The following table shows the 25 most significant pathways sorted by p-value from the select proteins in THP-1 Co-CM:

| Pathway name | Entities | | | | | Reactions | |
| --- | --- | --- | --- | --- | --- | --- | --- |
|  | **found** | **ratio** | **p-value** | **FDR*** | **found** | | **ratio** |
| **Microtubule-dependent trafficking of connexons from Golgi to the plasma membrane** | 3 / 20 | 0.001 | 4.64e-08 | 3.60e-06 | 1 / 2 | | 1.47e-04 |
| **Transport of connexons to the plasma membrane** | 3 / 21 | 0.001 | 5.37e-08 | 3.60e-06 | 1 / 3 | | 2.21e-04 |
| **RHO GTPases activate IQGAPs** | 3 / 40 | 0.002 | 3.70e-07 | 1.46e-05 | 1 / 5 | | 3.68e-04 |
| **Carboxyterminal post-translational modifications of tubulin** | 3 / 46 | 0.003 | 5.62e-07 | 1.46e-05 | 6 / 6 | | 4.42e-04 |
| **Activation of AMPK downstream of NMDARs** | 3 / 46 | 0.003 | 5.62e-07 | 1.46e-05 | 1 / 3 | | 2.21e-04 |
| **COPI-independent Golgi-to-ER retrograde traffic** | 3 / 83 | 0.005 | 3.28e-06 | 7.22e-05 | 2 / 7 | | 5.16e-04 |
| **Sealing of the nuclear envelope (NE) by ESCRT-III** | 3 / 91 | 0.005 | 4.32e-06 | 7.86e-05 | 1 / 7 | | 5.16e-04 |
| **Recruitment of NuMA to mitotic centrosomes** | 3 / 95 | 0.005 | 4.91e-06 | 7.86e-05 | 2 / 2 | | 1.47e-04 |
| **HSP90 chaperone cycle for steroid hormone receptors (SHR) in the presence of ligand** | 3 / 103 | 0.006 | 6.25e-06 | 8.75e-05 | 1 / 16 | | 0.001 |
| **Recycling pathway of L1** | 3 / 117 | 0.006 | 9.14e-06 | 1.19e-04 | 2 / 14 | | 0.001 |
| **Gap junction assembly** | 3 / 125 | 0.007 | 1.11e-05 | 1.31e-04 | 3 / 16 | | 0.001 |
| **Aggrephagy** | 3 / 128 | 0.007 | 1.19e-05 | 1.31e-04 | 4 / 15 | | 0.001 |
| **Gap junction trafficking** | 3 / 146 | 0.008 | 1.77e-05 | 1.76e-04 | 3 / 20 | | 0.001 |
| **Gap junction trafficking and regulation** | 3 / 154 | 0.008 | 2.07e-05 | 1.76e-04 | 3 / 24 | | 0.002 |
| **Kinesins** | 3 / 157 | 0.009 | 2.19e-05 | 1.76e-04 | 2 / 14 | | 0.001 |
| **Translocation of SLC2A4 (GLUT4) to the plasma membrane** | 3 / 171 | 0.009 | 2.83e-05 | 2.08e-04 | 1 / 15 | | 0.001 |
| **COPI-dependent Golgi-to-ER retrograde traffic** | 3 / 181 | 0.01 | 3.35e-05 | 2.08e-04 | 2 / 11 | | 8.10e-04 |
| **Resolution of Sister Chromatid Cohesion** | 3 / 186 | 0.01 | 3.63e-05 | 2.08e-04 | 4 / 8 | | 5.89e-04 |
| **EML4 and NUDC in mitotic spindle formation** | 3 / 187 | 0.01 | 3.69e-05 | 2.08e-04 | 2 / 5 | | 3.68e-04 |
| **RHO GTPases Activate Formins** | 3 / 188 | 0.01 | 3.75e-05 | 2.08e-04 | 1 / 27 | | 0.002 |
| **MHC class II antigen presentation** | 3 / 196 | 0.011 | 4.24e-05 | 2.08e-04 | 1 / 26 | | 0.002 |
| **Formation of tubulin folding intermediates by CCT/TriC** | 2 / 26 | 0.001 | 4.27e-05 | 2.08e-04 | 2 / 2 | | 1.47e-04 |
| **The role of GTSE1 in G2/M progression after G2 checkpoint** | 3 / 200 | 0.011 | 4.50e-05 | 2.08e-04 | 3 / 10 | | 7.37e-04 |
| **Separation of Sister Chromatids** | 3 / 205 | 0.011 | 4.85e-05 | 2.08e-04 | 2 / 8 | | 5.89e-04 |
| **Prefoldin mediated transfer of substrate to CCT/TriC** | 2 / 28 | 0.002 | 4.95e-05 | 2.08e-04 | 2 / 2 | | 1.47e-04 |

The following table shows the 25 most significant pathways sorted by p-value from the select proteins in A549 Mo/Co-CM:

| Pathway name | Entities | | | | | Reactions | |
| --- | --- | --- | --- | --- | --- | --- | --- |
|  | **found** | **ratio** | **p-value** | **FDR*** | **found** | | **ratio** |
| **Interleukin-4 and Interleukin-13 signaling** | 4 / 351 | 0.016 | 1.83e-04 | 0.022 | 2 / 47 | | 0.003 |
| **Crosslinking of collagen fibrils** | 2 / 24 | 0.001 | 1.90e-04 | 0.022 | 3 / 13 | | 9.58e-04 |
| **TP53 Regulates Transcription of Death Receptors and Ligands** | 2 / 40 | 0.002 | 5.23e-04 | 0.041 | 3 / 7 | | 5.16e-04 |
| **Elastic fibre formation** | 2 / 63 | 0.003 | 0.001 | 0.074 | 1 / 17 | | 0.001 |
| **Nucleotide salvage** | 2 / 71 | 0.003 | 0.002 | 0.075 | 3 / 22 | | 0.002 |
| **Assembly of collagen fibrils and other multimeric structures** | 2 / 79 | 0.004 | 0.002 | 0.078 | 3 / 26 | | 0.002 |
| **Chemokine receptors bind chemokines** | 2 / 102 | 0.005 | 0.003 | 0.109 | 2 / 19 | | 0.001 |
| **ATF4 activates genes in response to endoplasmic reticulum stress** | 2 / 136 | 0.006 | 0.006 | 0.139 | 1 / 7 | | 5.16e-04 |
| **Senescence-Associated Secretory Phenotype (SASP)** | 2 / 137 | 0.006 | 0.006 | 0.139 | 3 / 22 | | 0.002 |
| **PERK regulates gene expression** | 2 / 144 | 0.007 | 0.006 | 0.139 | 1 / 11 | | 8.10e-04 |
| **Neutrophil degranulation** | 3 / 480 | 0.022 | 0.007 | 0.139 | 4 / 10 | | 7.37e-04 |
| **Nucleotide catabolism** | 2 / 154 | 0.007 | 0.007 | 0.139 | 3 / 63 | | 0.005 |
| **Peptide ligand-binding receptors** | 3 / 513 | 0.024 | 0.009 | 0.15 | 4 / 77 | | 0.006 |
| **Interleukin-10 signaling** | 2 / 175 | 0.008 | 0.009 | 0.15 | 1 / 15 | | 0.001 |
| **Interaction with Cumulus Cells and the Zona Pellucida** | 1 / 14 | 6.53e-04 | 0.012 | 0.175 | 1 / 2 | | 1.47e-04 |
| **Collagen formation** | 3 / 207 | 0.01 | 0.013 | 0.18 | 5 / 77 | | 0.006 |
| **Metal sequestration by antimicrobial proteins** | 1 / 19 | 8.87e-04 | 0.016 | 0.206 | 1 / 5 | | 3.68e-04 |
| **Alternative complement activation** | 1 / 20 | 9.33e-04 | 0.017 | 0.217 | 7 / 9 | | 6.63e-04 |
| **Metabolism of nucleotides** | 2 / 316 | 0.015 | 0.028 | 0.296 | 6 / 141 | | 0.01 |
| **Pyrimidine salvage** | 1 / 36 | 0.002 | 0.03 | 0.296 | 1 / 10 | | 7.37e-04 |
| **Activation of C3 and C5** | 1 / 41 | 0.002 | 0.034 | 0.296 | 2 / 3 | | 2.21e-04 |
| **Activation of BMF and translocation to mitochondria** | 1 / 42 | 0.002 | 0.035 | 0.296 | 2 / 2 | | 1.47e-04 |
| **Fibronectin matrix formation** | 1 / 44 | 0.002 | 0.036 | 0.296 | 2 / 3 | | 2.21e-04 |
| **Purine salvage** | 1 / 45 | 0.002 | 0.037 | 0.296 | 2 / 12 | | 8.84e-04 |
| **Class A/1 (Rhodopsin-like receptors)** | 3 / 912 | 0.043 | 0.039 | 0.296 | 4 / 160 | | 0.012 |

The following table shows the 25 most significant pathways sorted by p-value from the select proteins in THP-1 Mo/Co-CM:

| Pathway name | Entities | | | | | Reactions | |
| --- | --- | --- | --- | --- | --- | --- | --- |
|  | **found** | **ratio** | **p-value** | **FDR*** | **found** | | **ratio** |
| **RUNX1 regulates genes involved in megakaryocyte differentiation and platelet function** | 2 / 388 | 0.018 | 0.002 | 0.03 | 5 / 33 | | 0.002 |
| **IRAK1 recruits IKK complex** | 1 / 16 | 7.47e-04 | 0.003 | 0.03 | 1 / 4 | | 2.95e-04 |
| **IRAK1 recruits IKK complex upon TLR7/8 or 9 stimulation** | 1 / 16 | 7.47e-04 | 0.003 | 0.03 | 1 / 4 | | 2.95e-04 |
| **Intracellular metabolism of fatty acids regulates insulin secretion** | 1 / 17 | 7.93e-04 | 0.003 | 0.03 | 1 / 2 | | 1.47e-04 |
| **JNK (c-Jun kinases) phosphorylation and activation mediated by activated human TAK1** | 1 / 26 | 0.001 | 0.005 | 0.03 | 1 / 3 | | 2.21e-04 |
| **TRAF6 mediated IRF7 activation in TLR7/8 or 9 signaling** | 1 / 27 | 0.001 | 0.005 | 0.03 | 1 / 5 | | 3.68e-04 |
| **activated TAK1 mediates p38 MAPK activation** | 1 / 27 | 0.001 | 0.005 | 0.03 | 1 / 5 | | 3.68e-04 |
| **Defective B3GALTL causes PpS** | 1 / 39 | 0.002 | 0.007 | 0.036 | 1 / 1 | | 7.37e-05 |
| **TICAM1, RIP1-mediated IKK complex recruitment** | 1 / 40 | 0.002 | 0.007 | 0.037 | 1 / 3 | | 2.21e-04 |
| **O-glycosylation of TSR domaincontaining proteins** | 1 / 41 | 0.002 | 0.008 | 0.038 | 2 / 2 | | 1.47e-04 |
| **Fibronectin matrix formation** | 1 / 44 | 0.002 | 0.008 | 0.041 | 2 / 3 | | 2.21e-04 |
| **IKK complex recruitment mediated by RIP1** | 1 / 45 | 0.002 | 0.008 | 0.042 | 1 / 3 | | 2.21e-04 |
| **Scavenging by Class B Receptors** | 1 / 45 | 0.002 | 0.008 | 0.042 | 1 / 5 | | 3.68e-04 |
| **SMAC (DIABLO) binds to IAPs** | 1 / 61 | 0.003 | 0.011 | 0.045 | 1 / 4 | | 2.95e-04 |
| **SMAC, XIAP-regulated apoptotic response** | 1 / 62 | 0.003 | 0.012 | 0.045 | 4 / 10 | | 7.37e-04 |
| **Activation of caspases through apoptosome-mediated cleavage** | 1 / 63 | 0.003 | 0.012 | 0.045 | 2 / 2 | | 1.47e-04 |
| **Transcriptional regulation by RUNX1** | 2 / 1,079 | 0.05 | 0.014 | 0.045 | 5 / 132 | | 0.01 |
| **IRAK2 mediated activation of TAK1 complex** | 1 / 78 | 0.004 | 0.014 | 0.045 | 1 / 5 | | 3.68e-04 |
| **Alpha-protein kinase 1 signaling pathway** | 1 / 80 | 0.004 | 0.015 | 0.045 | 2 / 10 | | 7.37e-04 |
| **TICAM1,TRAF6-dependent induction of TAK1 complex** | 1 / 80 | 0.004 | 0.015 | 0.045 | 1 / 6 | | 4.42e-04 |
| **Diseases associated with Oglycosylation of proteins** | 1 / 80 | 0.004 | 0.015 | 0.045 | 1 / 9 | | 6.63e-04 |
| **IRAK2 mediated activation of TAK1 complex upon TLR7/8 or 9**  **stimulation** | 1 / 84 | 0.004 | 0.016 | 0.046 | 1 / 5 | | 3.68e-04 |
| **TRAF6-mediated induction of TAK1 complex within TLR4 complex** | 1 / 85 | 0.004 | 0.016 | 0.046 | 1 / 6 | | 4.42e-04 |
| **Regulated proteolysis of p75NTR** | 1 / 94 | 0.004 | 0.017 | 0.046 | 1 / 5 | | 3.68e-04 |
| **Free fatty acids regulate insulin secretion** | 1 / 104 | 0.005 | 0.019 | 0.046 | 1 / 7 | | 5.16e-04 |
